# The accuracy of intraocular lens power calculation formulas based on artificial intelligence in highly myopic eyes: a systematic review and network meta-analysis

**DOI:** 10.3389/fpubh.2023.1279718

**Published:** 2023-11-09

**Authors:** Yi Zhou, Minhui Dai, Lingyu Sun, Xiangyi Tang, Ling Zhou, Zhiyao Tang, Jian Jiang, Xiaobo Xia

**Affiliations:** ^1^Eye Center of Xiangya Hospital, Central South University, Changsha, Hunan, China; ^2^Hunan Key Laboratory of Ophthalmology, Central South University, Changsha, Hunan, China; ^3^National Clinical Research Center for Geriatric Disorders, Xiangya Hospital, Central South University, Changsha, Hunan, China; ^4^Xiangya School of Nursing, Central South University, Changsha, Hunan, China

**Keywords:** intraocular lens, formulas, high myopia, artificial intelligence, prediction error

## Abstract

**Objective:**

To systematically compare and rank the accuracy of AI-based intraocular lens (IOL) power calculation formulas and traditional IOL formulas in highly myopic eyes.

**Methods:**

We screened PubMed, Web of Science, Embase, and Cochrane Library databases for studies published from inception to April 2023. The following outcome data were collected: mean absolute error (MAE), percentage of eyes with a refractive prediction error (PE) within ±0.25, ±0.50, and ±1.00 diopters (D), and median absolute error (MedAE). The network meta-analysis was conducted by R 4.3.0 and STATA 17.0.

**Results:**

Twelve studies involving 2,430 adult myopic eyes (with axial lengths >26.0 mm) that underwent uncomplicated cataract surgery with mono-focal IOL implantation were included. The network meta-analysis of 21 formulas showed that the top three AI-based formulas, as per the surface under the cumulative ranking curve (SUCRA) values, were XGBoost, Hill-RBF, and Kane. The three formulas had the lowest MedAE and were more accurate than traditional vergence formulas, such as SRK/T, Holladay 1, Holladay 2, Haigis, and Hoffer Q regarding MAE, percentage of eyes with PE within ±0.25, ±0.50, and ±1.00 D.

**Conclusions:**

The top AI-based formulas for calculating IOL power in highly myopic eyes were XGBoost, Hill-RBF, and Kane. They were significantly more accurate than traditional vergence formulas and ranked better than formulas with Wang–Koch AL modifications or newer generations of formulas such as Barrett and Olsen.

**Systematic review registration:**

https://www.crd.york.ac.uk/PROSPERO/, identifier: CRD42022335969.

## 1. Introduction

Myopia is a common refractive error that affects a significant proportion of the world population. The global prevalence of myopia was estimated to be 22.9% in 2000 and is projected to increase to 49.8% by 2050 (1). High myopia, defined as a refractive error of −6.00 diopters (D), is associated with axial lengths >26.0 mm (2). Myopia has been shown to be a risk factor for the development of cataracts, particularly nuclear cataracts and posterior subcapsular cataracts (3). Traditionally, vergence formulas such as SRK/T, Holladay 1 and 2, and Hoffer Q have been commonly used (4). However, these traditional formulas tend to result in hyperopic surprises, leading surgeons to empirically aim for a myopic target (5–7). Highly myopic eyes have complex structural changes such as zonular weakness (8), increases in anterior chamber depth (ACD) (9), premature vitreous degeneration, and posterior scleral staphyloma (6), which reduces the predictive accuracies of existing formulas. The Wang—Koch (WK) axial length (AL) adjustment (6, 10) and newer generations of formulas such as Barrett (available at: https://calc.apacrs.org/barrett_universal2105/) and Olsen (11) were developed to address these issues.

In recent years, artificial intelligence (AI) technology has been adopted to improve the accuracy and precision of IOL power calculations in myopic eyes. The Hill-radial basis function (RBF) formula (available at: http://rbfcalculator.com/online/index.html) and Kane formula (available at: www.iolformula.com) are gaining increasing popularity. Both formulas were developed and validated using large datasets. They used machine learning algorithms based on several patient-specific factors, including AL, keratometry (K), and lens thickness (LT). Other AI-based formulas, such as Emmetropia Verifying Optical (EVO) and Ladas Super Formula, have also been developed (11, 12).

Recent studies have compared the accuracy of AI-based formulas, traditional vergence formulas, newer generations of vergence formulas, and formulas with Wang–Koch adjustments. However, due to the large number of formula types, the process of recalculating IOL power using all the methods was time-consuming, and few studies have performed comprehensive comparisons between formulas. Our network meta-analysis, therefore, aims to comprehensively compare and rank the formulas in myopic patients who underwent cataract surgery. The findings of the present study will provide valuable clinical guidance for selecting the appropriate IOL formulas for myopic eyes.

## 2. Materials and methods

The present study was registered at Prospero (CRD42022335969, https://www.crd.york.ac.uk/PROSPERO/).

### 2.1. Search strategy and selection criteria

Two investigators (YZ and LS) searched PubMed, Web of Science, Embase, and Cochrane Library for studies published from their inception to 5 April 2023. The search terms used for searching the clinical condition are as follows: “myopia,” “long axial length,” “long AL,” “long eye,” “intraocular lens,” and “IOL.” The two investigators independently evaluated the title and abstract of all the identified studies. Additionally, we manually examined the reference lists of clinical trials, related meta-analyses, and systematic reviews to identify relevant studies.

Studies were retained if they met the following inclusion criteria: (1) focused on individuals with ocular AL longer than 26.0 mm; (2) included eyes with uncomplicated cataract surgery with in-the-bag fixated mono-focal IOL implantation; and (3) used at least two of the selected IOL power calculation formulas. Articles were excluded if they (1) used no AI formula; (2) included patients under 18 years; (3) had a history of other ocular diseases, eye surgery, or trauma; (4) included toric, multi-focal, piggyback, or not in-the-bag fixated IOL implantation; (5) included astigmatism correction using femtosecond laser-assisted cataract surgery; (6) did not provide any of the outcome data (MAE ± SD, percentage of eyes with a refractive PE within ±0.25, ±0.50, and ±1.00 D, MedAE); (7) measured optical biometry using approaches other than Lenstar, IOL Master, or Pentacam; and (8) were review articles or discussion papers, conference abstracts, or studies done on animals.

### 2.2. Data collection and processing

Two authors (MD and XT) extracted the following outcome data independently: (1) The percentage of eyes with a refractive prediction error (PE) within ±0.50 and ±1.00 diopters (D), (2) mean absolute error (MAE), and (3) median absolute error (MedAE) in refractive prediction. Participant and intervention characteristics were also extracted. For data that were missing or could not be directly obtained, we contacted the authors or used the WebPlotDigitizer tool (https://automeris.io/WebPlotDigitizer/) to read data from figures.

The percentage of eyes with PE within ±0.25, ±0.50, and ±1.00 D was dichotomous data. Thus, a binomial model was applied, and odds ratio (OR) with 95% CIs was calculated for the relative effect. The MAE was continuous data. Thus, a continuous model was applied, and a mean difference with 95% CIs was calculated for the relative effect. It is notable that MedAE was not suitable for the meta-analysis; therefore, only descriptive analyses were performed.

### 2.3. Quality assessment

Two authors (LZ and ZT) assessed the risk of bias in the included studies following the guidance of the quality appraisal tool for case series studies using a modified Delphi technique developed by the Institute of Health Economics (13). The following eight domains in the included studies were evaluated: study objective, study population, intervention and co-intervention, outcome measure, statistical analysis, results and conclusions, competing interests and sources of support, and new item. The clarity of each item in the eight domains was classified as “Yes,” “No,” and “Unclear/Partly stated.”

### 2.4. Publication bias detection

To assess the publication bias across studies, a graphic tool was developed by Chai (14). The code was integrated into an R package *netmeta*. The command *funnel()* generated a funnel plot to visualize publication bias across included studies. The obvious publication was presented as an asymmetric distribution of comparison-adjusted funnel plots.

### 2.5. Sensitivity analysis and inconsistency assessment

A sensitivity analysis was performed by repeating the network meta-analysis with the previously excluded high-risk studies. If the result was significantly influenced, the inconsistency between direct and indirect comparisons was assessed using the node-splitting approach (15), which differentiates direct and indirect evidence on a particular comparison and the design-by-treatment interaction models, assuming consistency throughout the entire network. A *p*-value < 0.05 was considered an inconsistency.

### 2.6. Surface under the cumulative ranking curve (SUCRA)

The probability of interventions at each ranking could be evaluated by SUCRA (16). The SUCRA value of each formula was assessed for the following primary outcomes: the percentage of eyes with a refractive PE within ±0.50 and ±1.00 D, MAE ± SD, and MedAE. A SUCRA value ranges from 0 to 100%, with a value closer to 100% indicating a higher likelihood that a formula is in the top rank. A SUCRA ranking figure was presented to report the SUCRA value for respective outcomes.

### 2.7. Subgroup analysis

To further compare the accuracy of AI-based formulas and conventional formulas, we performed subgroup analysis stratified by ALs (26.0–28.0, 28.0–30.0, and ≥30.0 mm) in studies where subgroup stratification with the same criteria was also conducted. Eyes with ALs >28.0 mm were defined as extremely myopic eyes. The MAE was compared in each subgroup using the evaluation metrics described above.

### 2.8. Statistical analysis

Network meta-analyses were performed using a random-effects model. All analyses were conducted using R 4.3.0 and STATA 17.0 for statistical analyses. The R packages *gemtc, ggplot2, netmeta, and ggrepel* were used for analysis, data output, and visualization.

## 3. Results

### 3.1. Study selection

The literature search strategy is presented in Supplementary Table S1. After removing duplications, 871 articles were identified from the literature search. Twenty-four full-text articles were further screened for eligibility. The preferred reporting items for systematic reviews and meta-analyses (PRISMA) flow diagram is shown in Supplementary Figure S1. The final inclusion of this systematic review consisted of 12 studies involving 2,430 adult myopic eyes that underwent uncomplicated cataract surgery with in-the-bag fixated mono-focal IOL implantation.

### 3.2. Study characteristics and network geometry

A summary of all eligible studies is shown in Supplementary Table S2 (17–28). The included AI formulas were Kane, Ladas super formula, Hill-RBF Version 2.0 and 3.0, XGBoost, K6, and Olsen. The included traditional formulas (based on vergence or ray-tracing) were Barrett Universal II, SRK/T, Holladay 1, Holladay 2, Hoffer Q, Haigis, Emmetropia Verifying Optical (EVO), and Olsen. If Wang–Koch (WK) adjustment was applied, the formula was analyzed as an independent formula. Table 1 shows the brief description and abbreviations for the formulas. The number of studies and eyes involved in each formula is shown in Supplementary Table S3.

**Table 1:** Brief description of the formulas included in the network meta-analysis.

**Formula**	**Classification**	**Year**	**Brief description**
Kane	AI-based	2016	Based on AL, K, ACD, LT (optional), CCT (optional), Gender, A constant, and post-operative refractive target. Blended approach (AI, regression, and vergence)
Ladas super formula	AI-based	2015	Applies most ideal calculations from other formulas (SRK/T, Hoffer Q, Holladay 1, Holladay, Haigis, etc.)
Hill-RBF			Based on AL, K, ACD, LT (optional), WTW (optional), CCT (optional), A-constant, and post-operative refractive target
Version 2.0	AI-based	2018	Excludes out-of-bounds values Might be significantly influenced by LT
Version 3.0	AI-based	2020	
All	AI-based	2018	Version 2.0 with out-of-bounds values
XGBoost	AI-based	2020	Based on the XGBoost machine learning regression technique. Incorporates several clinical features, and the BUII formula results Targets highly or extremely myopic eyes
K6	AI-based	2020	Transforms the optical biometer's AL to be the distance from the anterior cornea to the retinal pigment epithelium Uses a proprietary estimated lens position calculation based on post-operative measurement of 245 eyes
FullMonte IOL	AI-based		Uses a Monte Carlo Markov Chain simulator to produce its refractive predictions
Olsen	Traditional	2014	Ray-tracing Based on ACD, LT, and post-operative refractive target
Barrett Universal II	Traditional	2010	Based on AL, K, ACD, LT (optional), WTW (optional), LF/DF, A-constant, and post-operative refractive target The formula is not publicly available
**SRK/T**			
SRK/T	Traditional	1990	Based on AL, K, A-constant, and post-operative refractive target
SRK/T_WK	Traditional		SRK/T formula with WK adjustment
SRK/T_MWK	Traditional		SRK/T formula with modified WK adjustment
**Holladay 1**			
Holladay 1	Traditional	1988	Based on AL, K, SF, and post-operative refractive target
Holladay 1_WK	Traditional		Holladay 1 formula with WK adjustment
Holladay 1_MWK	Traditional		Holladay 1 formula with modified WK adjustment
Holladay 2	Traditional	1995	Based on AL, K, ACD, LT, WTW, CCT, Age, A-constant/ACD/SF, and post-operative refractive target
Hoffer Q	Traditional	1993	Based on AL, K, pACD, and post-operative refractive target
**Haigis**			
Haigis	Traditional	2004	Based on AL, K, ACD, three constants (a0; a1, which is associated with measured ACD; and a2, which is associated with measured AL), and post-operative refractive target
Haigis_WK	Traditional		Haigis formula with WK adjustment
Emmetropia Verifying Optical (EVO)	Traditional	2019	Based on AL, K, ACD, A-constant, LT (optional), CCT (optional), corneal refractive LVC status, and post-operative refractive target

The number of formulas involved in the studies ranged from 2 to 11. Of the included studies, 10 (83.3%) were from China, 1 (8.3%) recruited participants from countries in Europe, and 1 (8.3%) from Australia.

### 3.3. Risk of bias

The risk of bias from within the included articles is shown in Supplementary Table S4. All studies gained “Yes” in “study objective,” “outcome measures,” “statistic analysis,” and “competing interests and sources of support.” In the domain of “study population,” all 12 studies obtained at least three “Yes” responses. In “results and conclusions,” seven out of 12 studies gained over three “Yes” remarks. All the studies were retrospective designs. Overall, all studies gained at least 13 “Yes” responses among 20 items and were regarded as high quality.

Publication bias across studies was evaluated by funnel plot shown in Supplementary Figures S2–S5. The estimates of all the comparisons were symmetrically distributed in comparison-adjusted funnel plots, suggesting no publication bias across studies.

### 3.4. Mean absolute error in refractive prediction

**Figure 1:**
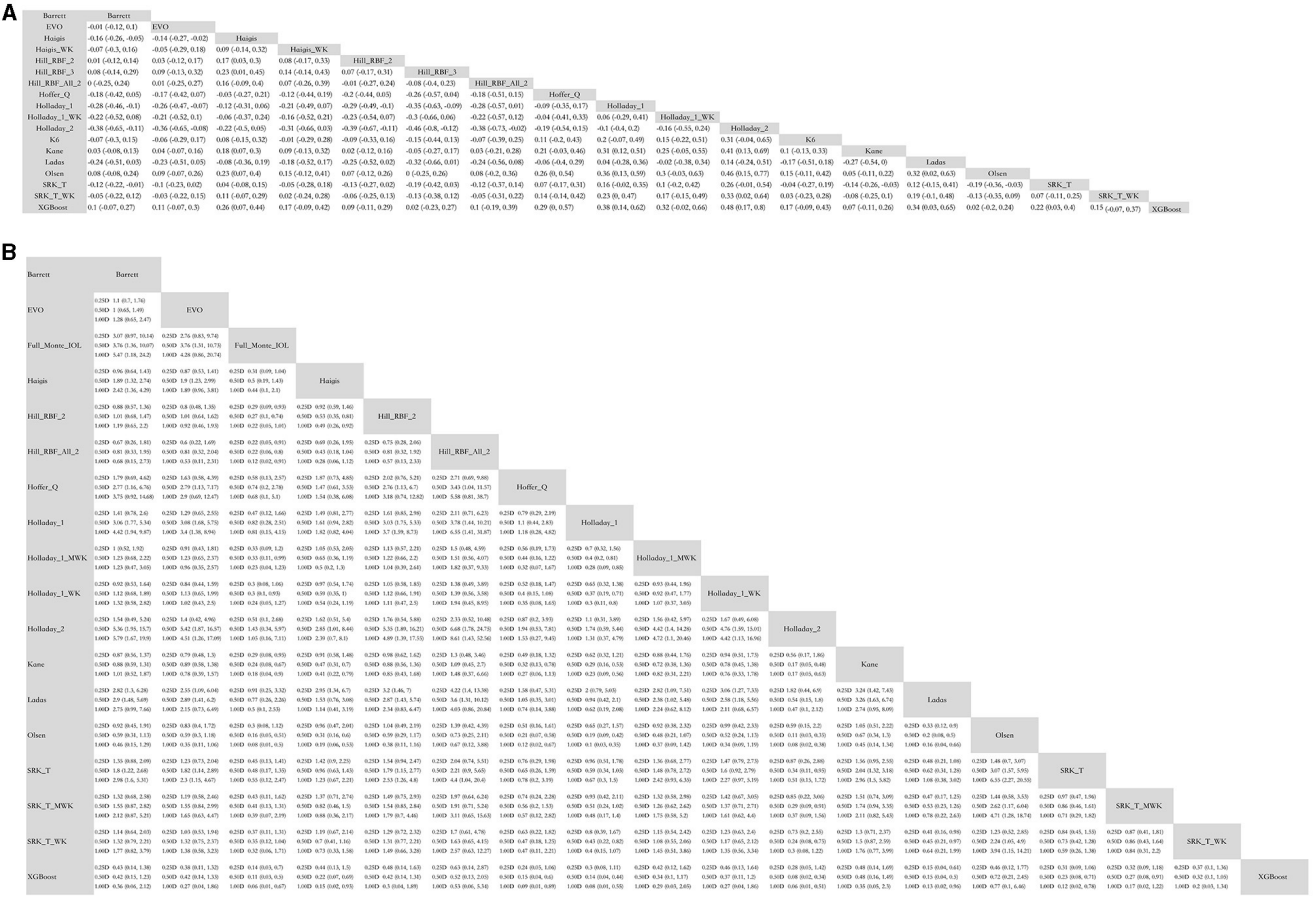
Mixed comparison of AI-based formulas and traditional formulas. **(A)** Mean absolute error. **(B)** Percentage of eyes with predictive error within ±0.25D, ±0.50 D, and ±1.00 D. WK, Wang–Koch AL adjustment; MWK, modified Wang–Koch AL adjustment; EVO, Emmetropia Verifying Optical; Barrett, Barrett Universal II; Ladas, Ladas super formula; RBF, radial basis function.

Mixed comparisons for MAE between AI-based formulas and traditional formulas are presented in Figure 1A. XGBoost formula was superior to Hoffer Q, and Kane was superior to SRK/T in terms of MAE. Most AI-based formulas, except for Kane, showed lower errors in refractive prediction when compared to Holladay 1. Hill-RBF Version 2 and 3 and Kane formulas showed lower errors when compared to Haigis. All AI-based formulas did not outperform Barrett, which is representative of the newer generation of traditional formulas. However, when Wang–Koch adjustment was applied to SRK/T, Haigis, and Holladay 1 formulas, there was no significant difference between traditional and AI-based formulas.

### 3.5. Percentage of eyes with a refractive PE within ±0.25D, ±0.50, and ±1.00 D

Mixed comparisons for the percentage of eyes with a PE within ±0.25, ±0.50, and ±1.00 D are presented in Figure 1B. In terms of the percentage of eyes with a PE within ±0.25 D (% PE within ±0.25 D), Kane was superior to Haigis, Hoffer Q, Holladay 1, and Holladay 2. Hill-RBF was better than Haigis, Hoffer Q, Holladay 1, Holladay 2, and SRK/T. However, if out-of-bounds were not excluded when applying Hill-RBF, the formula did not outperform SRK/T or Haigis. Ladas super formula showed the same percentage of eyes with a PE within ±0.25 D as all the traditional formulas. The XGBoost method was superior to most traditional formulas except for Wang–Koch adjusted formulas of newer generations.

Similarly, in terms of percentage PE within ±0.50 D, Kane and XGBoost were better than Haigis, Hoffer Q, Holladay 1, Holladay 2, and SRK/T. Hill-RBF was better than Hoffer Q, Holladay 1, Holladay 2, and SRK/T. However, Ladas super formula and FullMonte IOL formula were comparable to traditional ones.

Regarding the percentage of eyes with PE within ±1.00 D, XGBoost was superior to Haigis, Hoffer Q, Holladay 1, Holladay 2, and SRK/T. Kane and Hill-RBF were better than Haigis, Holladay 1, Holladay 2, and SRK/T. Again, Ladas super formula and FullMonte IOL formula were comparable to traditional ones.

It is notable that, in the percentage of eyes with PE within ±0.25, ±0.50, and ±1.00 D, AI-based formulas were comparable to newer generations of traditional vergence formulas or Wang–Koch adjusted formulas.

### 3.6. Median absolute error (MedAE) in refractive prediction

**Figure 2:**
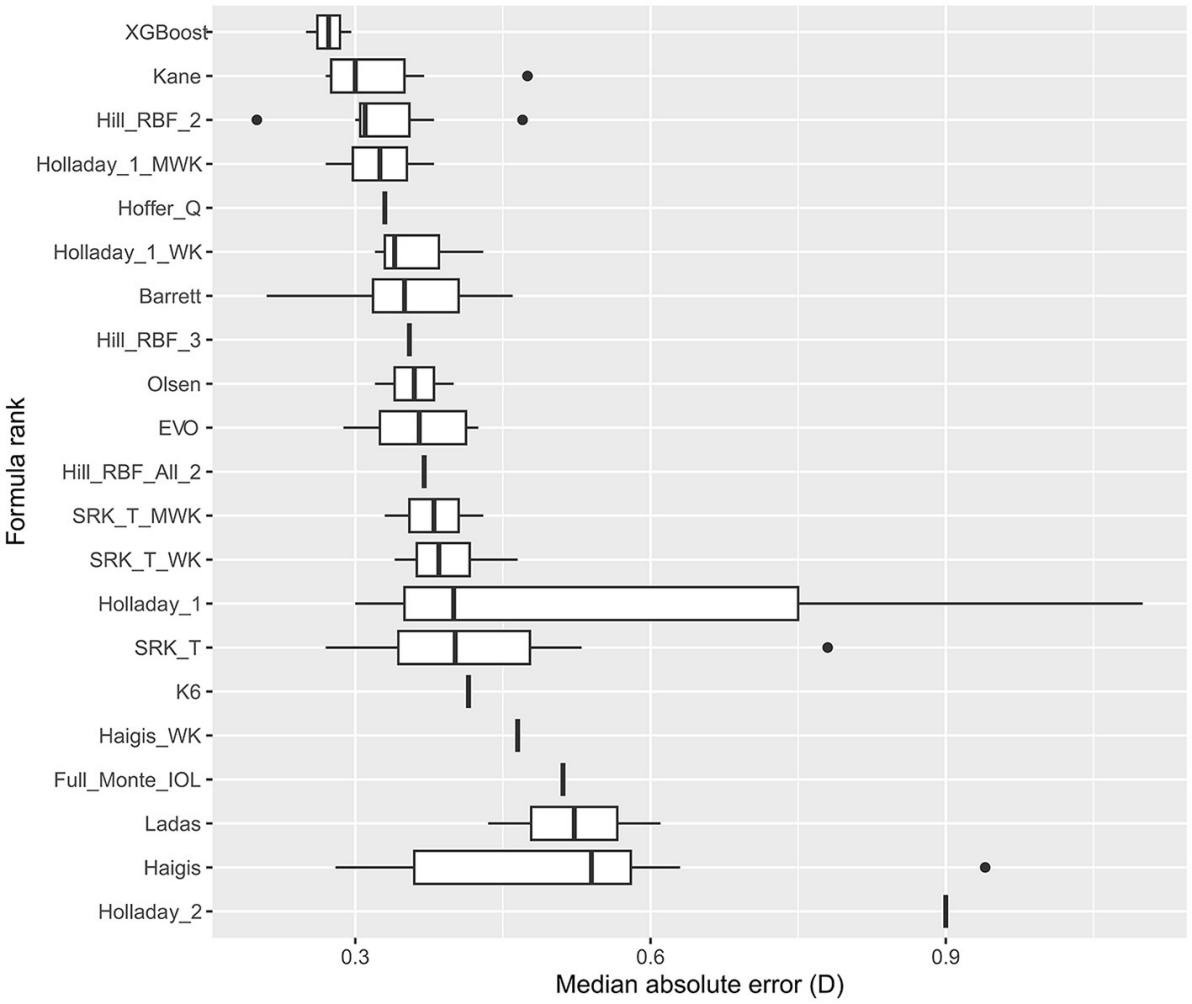
Formula rank in median absolute error (MedAE). WK, Wang–Koch AL adjustment; MWK, modified Wang–Koch AL adjustment; EVO, Emmetropia Verifying Optical; Barrett, Barrett Universal II; Ladas, Ladas super formula; RBF, radial basis function.

Figure 2 and Supplementary Table S5 show the analysis and formula ranking results for MedAE, and there were 12 studies in which 21 formulas were involved. We found that the XGBoost, Hill-BRF, and Kane formulas had lower MedAE (0.2730, 0.2730, and 0.2730, respectively).

### 3.7. SUCRA ranking of all outcomes

**Figure 3:**
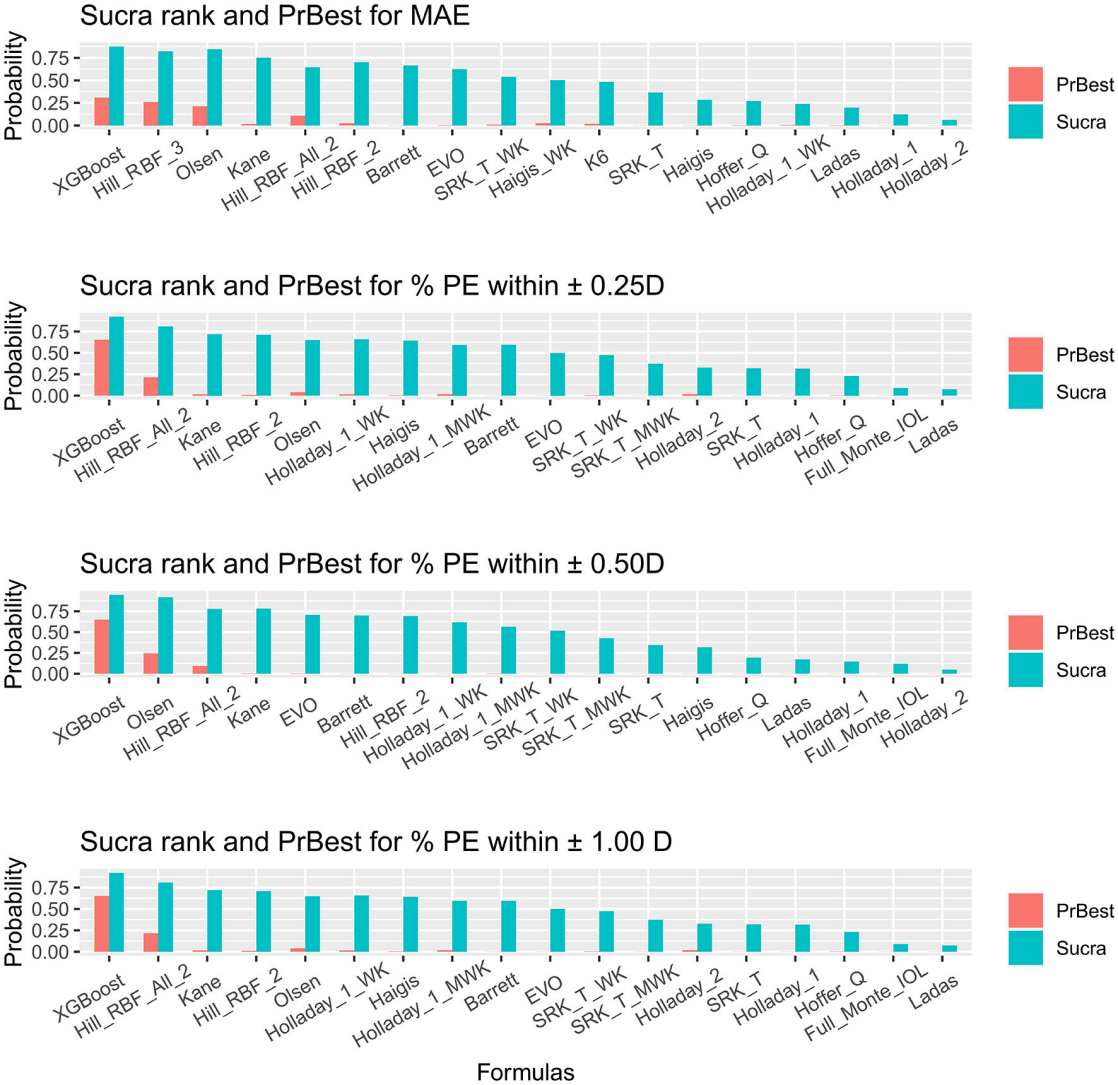
Surface under the cumulative ranking curve ranking plot. SUCRA, surface under the cumulative ranking curve; PrBest, probabilities of being the best; PE, predictive error; D, diopter; WK, Wang–Koch AL adjustment; MWK, modified Wang–Koch AL adjustment; EVO, Emmetropia Verifying Optical; Barrett, Barrett Universal II; Ladas, Ladas super formula; RBF, radial basis function.

The SUCRA values provided the probabilities of AI-based or traditional formulas at each ranking and are presented in Figure 3 and Supplementary Tables S6–S9. The probabilities of each formula being the best were also plotted.

For obtaining the minimal MAE, XGBoost, Hill-RBF Version 3.0, and Olsen ranked as the three best (Figure 3A). Holladay 2 ranked the worst. However, XGBoost did not show significant superiority to Hill-RBF Version 3.0 and Olsen [Hill_RBF_3 vs. XGBoost = 0.02 (−0.23, 0.27) vs. Olsen vs. XGBoost = 0.02 (−0.2, 0.24); Figure 1A]. The probabilities of XGBoost, Hill-RBF Version 3.0, and Olsen being the best were 0.30880, 0.26040, and 0.21345, respectively.

For the percentage of eyes with a PE within ±0.25 D, ±0.50 D, and ±1.00 D, XGBoost, Hill-RBF Version 2.0, and Kane were the best ranking AI-based formulas (Figure 3B). Similarly, there was no significant difference between each of the three formulas. Among all the formulas, Ladas had the lowest probability in the percentage of eyes with PE within ±0.25 D and ±1.00 D (both 0.000125), while Holladay 2 had the lowest probability in percentage PE within ±0.50 D (0.000000).

### 3.8. Inconsistency analysis

To detect the inconsistency within networks, the node-splitting approach was applied. No significant consistency (*p* > 0.05) was observed in terms of MAE or percentage of eyes with PE within ±0.25 D, ±0.50 D, and ±1.00 D (Supplementary Tables S10–S13). Significant consistency (*p* > 0.05) was detected in the analyses above.

### 3.9. Subgroup analysis

Six of the studies underwent subgroup analysis using the previously described criteria (stratifying ALs into three subgroups: 26.0–28.0, 28.0–30.0, and ≥30.0 mm, Supplementary Tables S14–S16). Three studies involving 11 formulas and 381 eyes were included for subgroup analysis because they provided comprehensive MAE values, SD values, and sample sizes required for network meta-analysis using continuous data.

**Figure 4:**
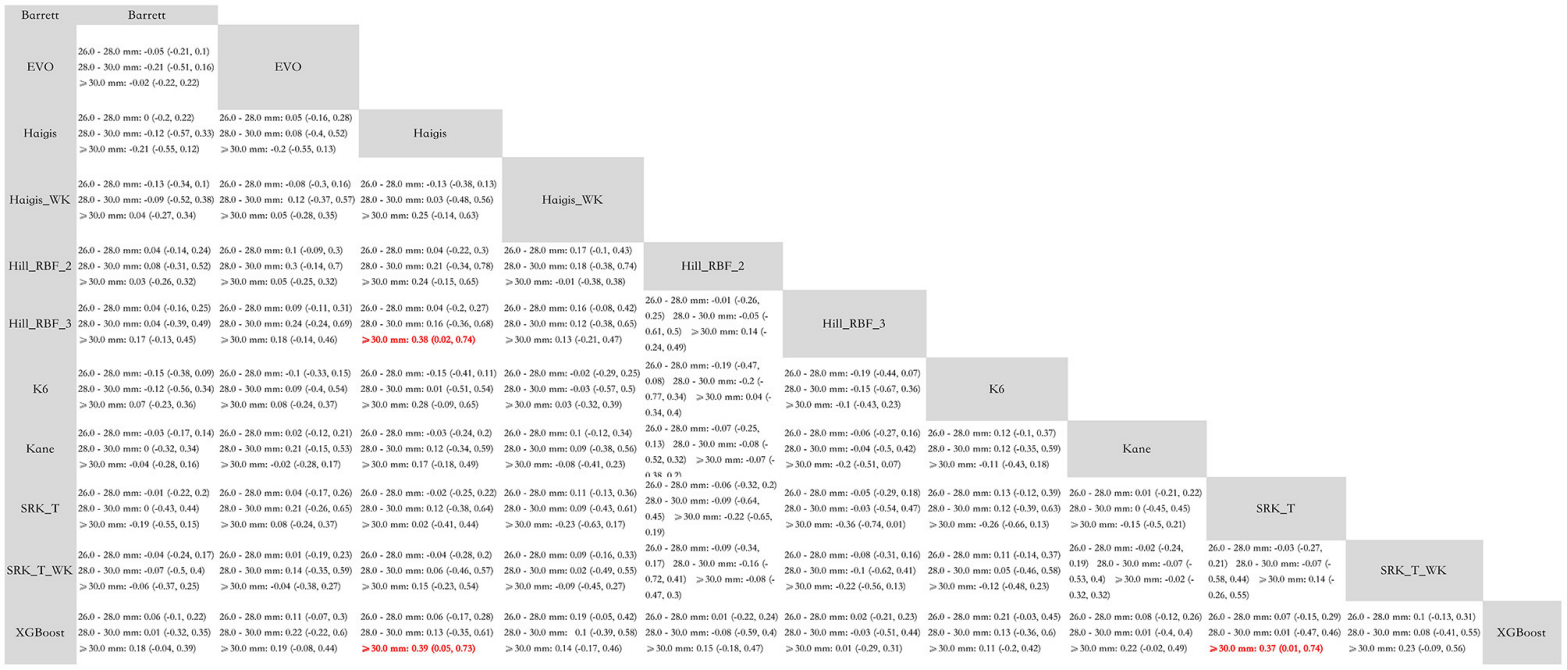
Mixed comparison of subgroup analysis stratified by axial lengths.

The mixed comparisons of the AI-based and conventional formulas for each subgroup are presented in Figure 4. In eyes with ALs between 26.0–28.0 and 28.0–30.0 mm, all formulas were comparable to each other. In extremely myopic eyes with an AL ≥30.0 mm, the XGBoost formula was significantly more accurate than Haigis [MAE decreased by 0.39 (0.05, 0.73)] and SRK/T [MAE decreased by 0.37 (0.01, 0.74)], and Hill-RBF 3.0 was significantly more accurate than Haigis [MAE decreased by 0.38 (0.02, 0.74)]. Other formulas were comparable to each other in the subgroup with ALs >30.0 mm.

The SUCRA values and the probabilities of each formula being the best are provided in Supplementary Table S17. In the 26.0–28.0 mm subgroup, XGBoost (SUCRA = 0.79861), Hill-RBF 2.0 (SUCRA = 0.756005), and Hill-RBF 3.0 (SUCRA = 0.717455) were the top three formulas. In the 28.0–30.0 mm subgroup, Hill-RBF 3.0 (SUCRA = 0.644415), XGBoost (SUCRA = 0.599635), and Kane (SUCRA = 0.583095) were the top three formulas. In the ≥30.0 mm subgroup, XGBoost (SUCRA = 0.88663), Hill-RBF 3.0 (SUCRA = 0.855355), and Hill-RBF 2.0 (SUCRA = 0.580605) were the top three formulas.

## 4. Discussion

The present study is the first network meta-analysis to evaluate the accuracy of AI-based formulas for IOL power calculation in myopic eyes with axial lengths >26.0 mm. To clearly discuss the characteristics of each formula, we divided the 21 formulas into the following four types: (1) AI-based formulas; (2) newer generation of traditional formulas (such as Barrett and Olsen); (3) traditional vergence formulas (such as SRK/T, Hoffer Q, Holladay 1, and Holladay 2); and (4) traditional vergence formulas with AL adjustment (such as SRK/T_WK or SRK/T_MWK). By analyzing MAE and the percentage of eyes with ±0.25 D, ±0.50 D, and ±1.00 D of prediction error, we demonstrated that XGBoost, Kane, and Hill-RBF were the most accurate AI-based formulas.

The XGBoost formula was designed exclusively for myopic eyes (19, 26). It was developed and validated using data from 1,450 patients. The subgroup analysis in our study showed that, in eyes with an AL ≥30.0 mm, the XGBoost formula was significantly more accurate than traditional ones (such as Haigis and SRK/T). This advantage might result from the study design where the average AL of the recruited patients was >29.00 mm, and extremely high myopic eyes were taken into consideration. Additionally, the XGBoost formula included cases where <-2.5 D myopic refractive targets were scheduled (19). It was, therefore, suggestive that the XGBoost formula might be more reliable for IOL power prediction in highly or extremely myopic eyes compared to other AI-based formulas. Most recently, the Zhu–Lu formula (https://HM-ZLF.com/), developed by the same team that developed using XGBoost and support vector regression (SVR) algorithms, demonstrated improved and stable accuracy compared to other formulas (29).

Hill-RBF was the first IOL power calculation method based purely on artificial intelligence and was installed on Lenstar (HaagStreit, Switzerland) (30). Hill-RBF 2.0 is based on more than 12,000 eyes and can calculate IOL power for a target different than zero. It was based on AL, K, ACD, and LT [CCT, LT, and CD are optional (31, 32)]. The Hill-RBF 2.0 was limited due to the “out-of-bounds” warnings. Hill-RBF 3.0 formula, an improvement from its 2.0 version, utilized pattern recognition and employed an advanced method of data interpolation. However, there has been no study directly comparing Hill-RBF 3.0 and 2.0 in eyes with an AL >26.0 mm. Tsessler et al. (32) found that Hill-RBF 3.0 was more accurate than Hill-RBF 2.0, though their study did not necessarily focus on myopic eyes. Network meta-analysis offers an indirect approach to comparing two formulas without actually conducting the comparative trial. The SUCRA ranking of MAE in our study showed that the Hill-RBF 3.0 ranked higher than the 2.0 version. Moreover, the subgroup analysis further demonstrated the superiority of Hill-RBF 3.0 in eyes with an AL >30.0 mm. Although the MedAE ranking in Figure 2 shows Hill-RBF 2.0 with a higher ranking than the 3.0 version, it is worth mentioning that MedAE was not suitable for meta-analysis (33). Therefore, only descriptive analyses were presented in the MedAE ranking, and no statistical analysis could be undertaken. Hill-RBF formulas were suitable for highly myopic eyes, and Hill-RBF 3.0 was particularly accurate for extremely myopic eyes.

The Kane formula is an unpublished one, and the structure is largely unknown. It is based on theoretical optics, contains some elements of artificial intelligence, and uses AL, K, ACD, and gender to predict the IOL position, with LT and CCT being optional factors (34, 35). The formula considers factors such as ACD and LT, which are known to affect IOL power calculations in myopic eyes. Our findings suggest that the Kane formula was more accurate than conventional formulas such as Haigis and SRK/T in highly myopic eyes. However, as the subgroup analysis suggested, Kane was comparable to traditional formulas when dealing with extremely myopic eyes.

The Ladas super formula was created by Dr. John Ladas and further optimized in 2019 using the post-operative data of more than 4,000 eyes (35). It uses a three-dimensional model to choose the best IOL formula among existing ones for a particular AL or corneal power (12). Ang et al. (36) found that, in myopic eyes, Ladas was less accurate than AI-based or newer formulas such as Kane and Barrett. They also demonstrated a strong positive correlation between absolute prediction error and AL with the Ladas and SRK/T formulas, especially in extremely long ALs. Similarly, data in our study showed that the Ladas formula ranked 16th out of the 18 formulas in the MAE SUCRA ranking analysis and was not superior to traditional formulas in other evaluation analyses. The reason for its unexpected poor performance was partially due to the fact that Ladas was developed by combining conventional formulas such as Hoffer Q, Holladay 1, Holladay 2 (with Wang–Koch adjustment) (6), and SRK/T formulas (11) rather than creating new algorithms as most recent AI formulas did. When using Ladas formula in highly myopic eyes, other formulas should also be used to choose the most accurate one. However, physicians should be careful when using the Ladas formula in eyes with an AL >30.0 mm.

The newer generation of formulas showed superiority over traditional vergence formulas. Barrett formula incorporates AL, K, ACD, LT, WTW, age, corneal power, and estimated lens position (37–39). The Olsen formula is characterized by the ray-tracing technique and the C constant concept (40–43). In the present study, both Barrett and Olsen showed no significant difference from AI-based formulas. However, the Olsen formula had significantly lower MAE than SRK/T, Haigis, Hoffer Q, Holladay 1, and Holladay 2. Barrett formula had significantly lower MAE than most traditional formulas except for Hoffer Q. In terms of the percentage of eyes within ±0.50 and ±1.00 D PE, Barrett and Olsen formulas showed significant superiority over traditional formulas. The Wang–Koch adjustment was developed to be applied in eyes with longer ALs that have IOL power calculation with the Holladay 1 formula (6, 10). In the present study, there was no significant difference between formulas with Wang–Koch adjustment and AI-based formulas in terms of MAE and percentage of eyes within ±0.25 D, ±0.50 D, and ±1.00 D PE.

## 5. Limitations and recommendations

This network meta-analysis has several limitations inherent to the methodology applied in the study. First, 10 out of the 12 studies included in this study were conducted in China, and the other two were from Australia and Europe. Therefore, the conclusions of our study might not be generalized to other populations. Second, one study included both eyes of some patients, and the correlation between eyes is a potential limitation of the analysis. Third, two studies (27, 28) used the lens constants from the User Group for Laser Interference Biometry (ULIB), and more research is needed to analyze the effect of ULIB in the future.

## 6. Conclusion

In summary, the overall evidence indicated that in cataract patients with ALs >26.0 mm, AI-based formulas (especially XGBoost, Hill-RBF, and Kane) were promising in obtaining lower MAE and a higher percentage of eyes within ±0.25 D, ±0.50 D, and ±1.00 D of prediction error when compared to traditional vergence formulas. AI-based formulas tended to perform better than newer generations of formulas (such as Barrett and Olsen) and formulas with Wang–Koch adjustment, but the superiority was not significant. In future studies, sufficiently sized and geographically dispersed studies are warranted to validate the effect of AI-based formulas.

## Data availability statement

The original contributions presented in the study are included in the article/Supplementary material, further inquiries can be directed to the corresponding authors.

## Author contributions

YZ: Conceptualization, Data curation, Formal analysis, Methodology, Software, Writing – original draft. MD: Conceptualization, Data curation, Writing – original draft. LS: Writing – review & editing. XT: Software, Writing – review & editing. LZ: Software, Writing – review & editing. ZT: Software, Writing – review & editing. JJ: Conceptualization, Supervision, Writing – review & editing. XX: Conceptualization, Funding acquisition, Project administration, Resources, Supervision, Writing – review & editing.

## References

[1] Holden BA Fricke TR Wilson DA Jong M Naidoo KS Sankaridurg P . Global prevalence of myopia and high myopia and temporal trends from 2000 through 2050. Ophthalmology. (2016) 123:1036–42. 10.1016/j.ophtha.2016.01.006 26875007

[2] Haigis W. Intraocular lens calculation in extreme myopia. J Cataract Refract Surg. (2009) 35:906–11. 10.1016/j.jcrs.2008.12.035 19393892

[3] Pan CW Cheng CY Saw SM Wang JJ Wong TY. Myopia and age-related cataract: a systematic review and meta-analysis. Am J Ophthalmol. (2013) 156:1021–33.e1. 10.1016/j.ajo.2013.06.005 23938120

[4] Voytsekhivskyy OV. Development and clinical accuracy of a new intraocular lens power formula (VRF) compared to other formulas. Am J Ophthalmol. (2018) 185:56–67. 10.1016/j.ajo.2017.10.020 29102605

[5] Hill DC Sudhakar S Hill CS King TS Scott IU Ernst BB . Intraoperative aberrometry versus preoperative biometry for intraocular lens power selection in axial myopia. J Cataract Refract Surg. (2017) 43:505–10. 10.1016/j.jcrs.2017.01.014 28532936

[6] Wang L Shirayama M Ma XJ Kohnen T Koch DD. Optimizing intraocular lens power calculations in eyes with axial lengths above 250 mm. J Cataract Refract Surg. (2011) 37:2018–27. 10.1016/j.jcrs.2011.05.042 22018365

[7] Abulafia A Barrett GD Rotenberg M Kleinmann G Levy A Reitblat O . Intraocular lens power calculation for eyes with an axial length greater than 260 mm: comparison of formulas and methods. J Cataract Refract Surg. (2015) 41:548–56. 10.1016/j.jcrs.2014.06.033 25708208

[8] Miyoshi T Fujie S Yoshida H Iwamoto H Tsukamoto H Oshika T. Effects of capsular tension ring on surgical outcomes of premium intraocular lens in patients with suspected zonular weakness. PLoS ONE. (2020) 15:e0228999. 10.1371/journal.pone.0228999 32092103 PMC7039513

[9] Ning X Yang Y Yan H Zhang J. Anterior chamber depth - a predictor of refractive outcomes after age-related cataract surgery. BMC Ophthalmol. (2019) 19:134. 10.1186/s12886-019-1144-8 31238910 PMC6591866

[10] Wang L Koch DD. Modified axial length adjustment formulas in long eyes. J Cataract Refract Surg. (2018) 44:1396–7. 10.1016/j.jcrs.2018.07.049 30274847

[11] Melles RB Kane JX Olsen T Chang WJ. Update on intraocular lens calculation formulas. Ophthalmology. (2019) 126:1334–5. 10.1016/j.ophtha.2019.04.011 30980854

[12] Ladas JG Siddiqui AA Devgan U Jun AS. A 3-D “super surface” combining modern intraocular lens formulas to generate a “super formula” and maximize accuracy. JAMA Ophthalmol. (2015) 133:1431–6. 10.1001/jamaophthalmol.2015.3832 26469147

[13] Lucas NP Macaskill P Irwig L Bogduk N. The development of a quality appraisal tool for studies of diagnostic reliability (QAREL). J Clin Epidemiol. (2010) 63:854–61. 10.1016/j.jclinepi.2009.10.002 20056381

[14] Chaimani A Higgins JP Mavridis D Spyridonos P Salanti G. Graphical tools for network meta-analysis in STATA. PLoS ONE. (2013) 8:e76654. 10.1371/journal.pone.0076654 24098547 PMC3789683

[15] van Valkenhoef G Dias S Ades AE Welton NJ. Automated generation of node-splitting models for assessment of inconsistency in network meta-analysis. Res Synth Methods. (2016) 7:80–93. 10.1002/jrsm.1167 26461181 PMC5057346

[16] Salanti G Ades AE Ioannidis JP. Graphical methods and numerical summaries for presenting results from multiple-treatment meta-analysis: an overview and tutorial. J Clin Epidemiol. (2011) 64:163–71. 10.1016/j.jclinepi.2010.03.016 20688472

[17] Bernardes J Raimundo M Lobo C Murta JN. A comparison of intraocular lens power calculation formulas in high myopia. J Refract Surg. (2021) 37:207–11. 10.3928/1081597X-20201123-01 34038295

[18] Wan KH Lam TCH Yu MCY Chan TCY. Accuracy and precision of intraocular lens calculations using the new hill-RBF version 20 in eyes with high axial myopia. Am J Ophthalmol. (2019) 205:66–73. 10.1016/j.ajo.2019.04.019 31078534

[19] Wei L Song Y He W Chen X Ma B Lu Y . Accuracy improvement of IOL power prediction for highly myopic eyes with an XGBoost machine learning-based calculator. Front Med. (2020) 7:592663. 10.3389/fmed.2020.592663 33425941 PMC7793738

[20] Kane JX Van Heerden A Atik A Petsoglou C. Accuracy of 3 new methods for intraocular lens power selection. J Cataract Refract Surg. (2017) 43:333–9. 10.1016/j.jcrs.2016.12.021 28410714

[21] Lin L Xu M Mo E Huang S Qi X Gu S . Accuracy of newer generation IOL power calculation formulas in eyes with high axial myopia. J Refract Surg. (2021) 37:754–8. 10.3928/1081597X-20210712-08 34756144

[22] Mo E Lin L Wang J Huo Q Yang Q Liu E . Clinical accuracy of 6 intraocular lens power calculation formulas in elongated eyes, according to anterior chamber depth. Am J Ophthalmol. (2022) 233:153–62. 10.1016/j.ajo.2021.07.017 34303685

[23] Guo C Yin S Qiu K. Zhang M. Comparison of accuracy of intraocular lens power calculation for eyes with an axial length greater than 290 mm. Int Ophthalmol. (2022) 42:2029–38. 10.1007/s10792-021-02194-1 35536455 PMC9085560

[24] Chen Y Wei L He W Lu Y Zhu X. Comparison of Kane, Hill-RBF 2.0, Barrett Universal II, and emmetropia verifying optical formulas in eyes with extreme myopia [published correction appears in J Refract Surg. 2022;38:474]. J Refract Surg. (2021) 37:680–5. 10.3928/1081597X-20210712-03 34661474

[25] Ji J Liu Y Zhang J Wu X Shao W Ma B . Comparison of six methods for the intraocular lens power calculation in high myopic eyes. Eur J Ophthalmol. (2021) 31:96–102. 10.1177/1120672119889016 31744328

[26] Wei L Cheng K He W Zhu X Lu Y. Application of total keratometry in ten intraocular lens power calculation formulas in highly myopic eyes. Eye Vis. (2022) 9:21. 10.1186/s40662-022-00293-3 35676698 PMC9178866

[27] Cheng H Wang L Kane JX Li J Liu L Wu M. Accuracy of artificial intelligence formulas and axial length adjustments for highly myopic eyes. Am J Ophthalmol. (2021) 223:100–7. 10.1016/j.ajo.2020.09.019 32950507

[28] Liu J Wang L Chai F Han Y Qian S Koch DD . Comparison of intraocular lens power calculation formulas in Chinese eyes with axial myopia. J Cataract Refract Surg. (2019) 45:725–31. 10.1016/j.jcrs.2019.01.018 31146930

[29] Guo D He W Wei L Song Y Qi J Yao Y . The Zhu-Lu formula: a machine learning-based intraocular lens power calculation formula for highly myopic eyes. Eye Vis. (2023) 10:26. 10.1186/s40662-023-00342-5 37259154 PMC10233923

[30] Kane JX Chang DF. Intraocular lens power formulas, biometry, and intraoperative aberrometry: a review. Ophthalmology. (2021) 128:e94–114. 10.1016/j.ophtha.2020.08.010 32798526

[31] Chung J Bu JJ Afshari NA. Advancements in intraocular lens power calculation formulas. Curr Opin Ophthalmol. (2022) 33:35–40. 10.1097/ICU.0000000000000822 34854826

[32] Tsessler M Cohen S Wang L Koch DD Zadok D Abulafia A. Evaluating the prediction accuracy of the Hill-RBF 30 formula using a heteroscedastic statistical method. J Cataract Refract Surg. (2022) 48:37–43. 10.1097/j.jcrs.0000000000000702 34016821

[33] Wen D Yu J Zeng Z McAlinden C Hu L Feng K . Network meta-analysis of no-history methods to calculate intraocular lens power in eyes with previous myopic laser refractive surgery. J Refract Surg. (2020) 36:481–90. 10.3928/1081597X-20200519-04 32644171

[34] Connell BJ Kane JX. Comparison of the Kane formula with existing formulas for intraocular lens power selection. BMJ Open Ophthalmol. (2019) 4:e000251. 10.1136/bmjophth-2018-000251 31179396 PMC6528763

[35] Savini G Taroni L Hoffer KJ. Recent developments in intraocular lens power calculation methods-update 2020. Ann Transl Med. (2020) 8:1553. 10.21037/atm-20-2290 33313298 PMC7729321

[36] Ang RET Rapista AJB Remo JTM Tan-Daclan MAT Cruz EM. Clinical outcomes and comparison of intraocular lens calculation formulas in eyes with long axial myopia. Taiwan J Ophthalmol. (2021) 12:305–11. 10.4103/tjo.tjo_7_21 36248093 PMC9558479

[37] Barrett GD. Intraocular lens calculation formulas for new intraocular lens implants. J Cataract Refract Surg. (1987) 13:389–96. 10.1016/S0886-3350(87)80037-8 3625516

[38] Barrett GD. An improved universal theoretical formula for intraocular lens power prediction. J Cataract Refract Surg. (1993) 19:713–20. 10.1016/S0886-3350(13)80339-2 8271166

[39] Koch DD Hill W Abulafia A Wang L. Pursuing perfection in intraocular lens calculations: I. Logical approach for classifying IOL calculation formulas. J Cataract Refract Surg. (2017) 43:717–8. 10.1016/j.jcrs.2017.06.006 28732602

[40] Olsen T. Theoretical approach to intraocular lens calculation using Gaussian optics. J Cataract Refract Surg. (1987) 13:141–5. 10.1016/S0886-3350(87)80128-1 3572769

[41] Olsen T Corydon L Gimbel H. Intraocular lens power calculation with an improved anterior chamber depth prediction algorithm. J Cataract Refract Surg. (1995) 21:313–9. 10.1016/S0886-3350(13)80140-X 7674170

[42] Olsen T. Prediction of the effective postoperative (intraocular lens) anterior chamber depth. J Cataract Refract Surg. (2006) 32:419–24. 10.1016/j.jcrs.2005.12.139 16631049

[43] Olsen T Hoffmann P. C constant: new concept for ray tracing-assisted intraocular lens power calculation. J Cataract Refract Surg. (2014) 40:764–73. 10.1016/j.jcrs.2013.10.037 24767910

