# Supplementary material for: The accuracy of intraocular lens power calculation formulas based on artificial intelligence in highly myopic eyes: a systematic review and network meta-analysis
Source: Front Public Health. 2023 Nov 9;11:1279718. doi: 10.3389/fpubh.2023.1279718 (PMC10670805; doi:10.3389/fpubh.2023.1279718)
Supplement: Supplementary file 2 [file Data_Sheet_1.docx]

**Supplemental Materials**


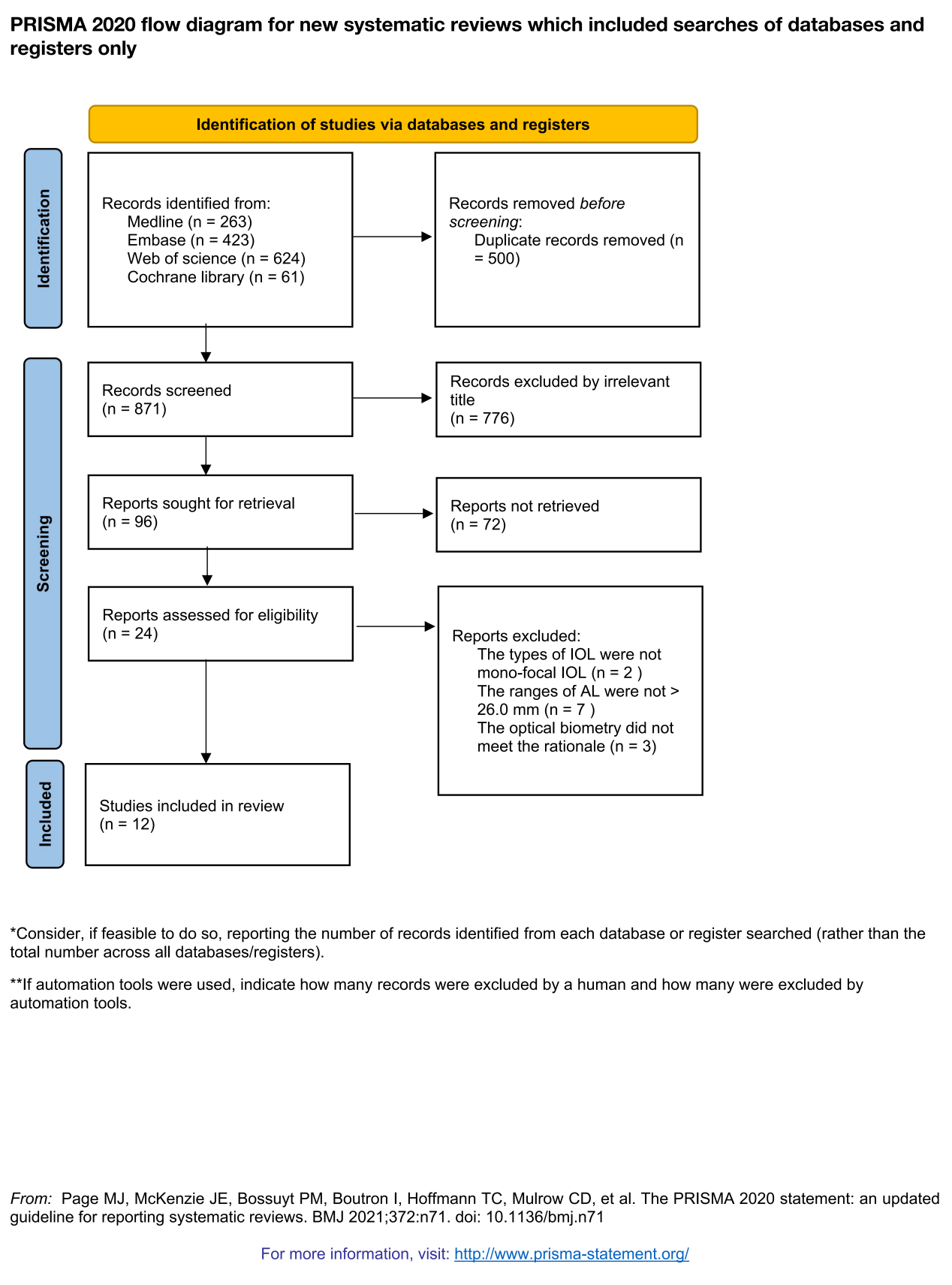


**Figure S1. Preferred reporting items for systematic reviews and meta-analyses (PRISMA) flow diagram.**


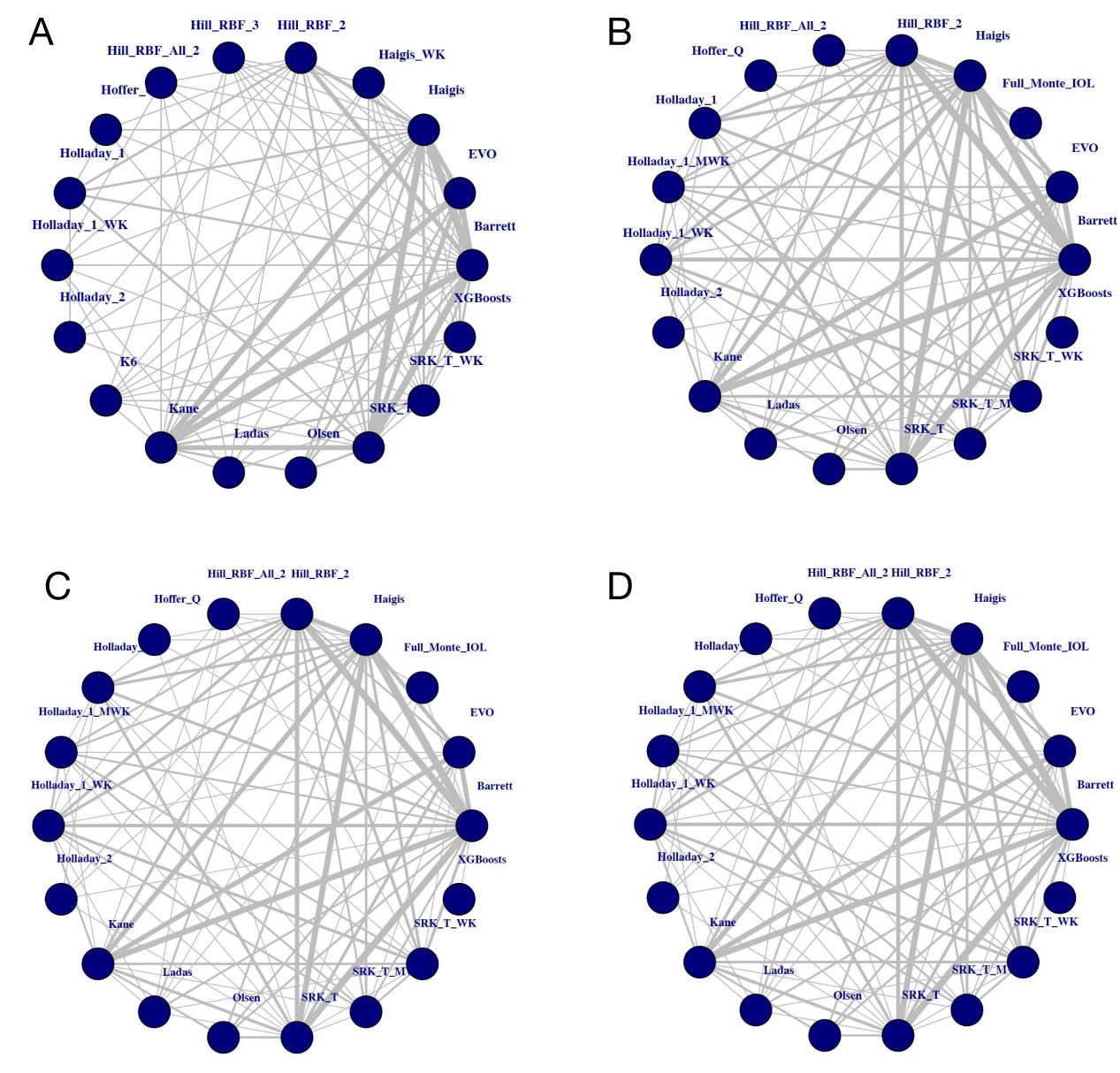


**Figure S2. Network of mixed comparison for the formulas.**

1. Mean absolute error. **(B)** Percentage of eyes with predictive error within ± 0.25D. **(C)** Percentage of eyes with predictive error within ± 0.50 D. **(D)** Percentage of eyes with predictive error within ± 1.00 D.


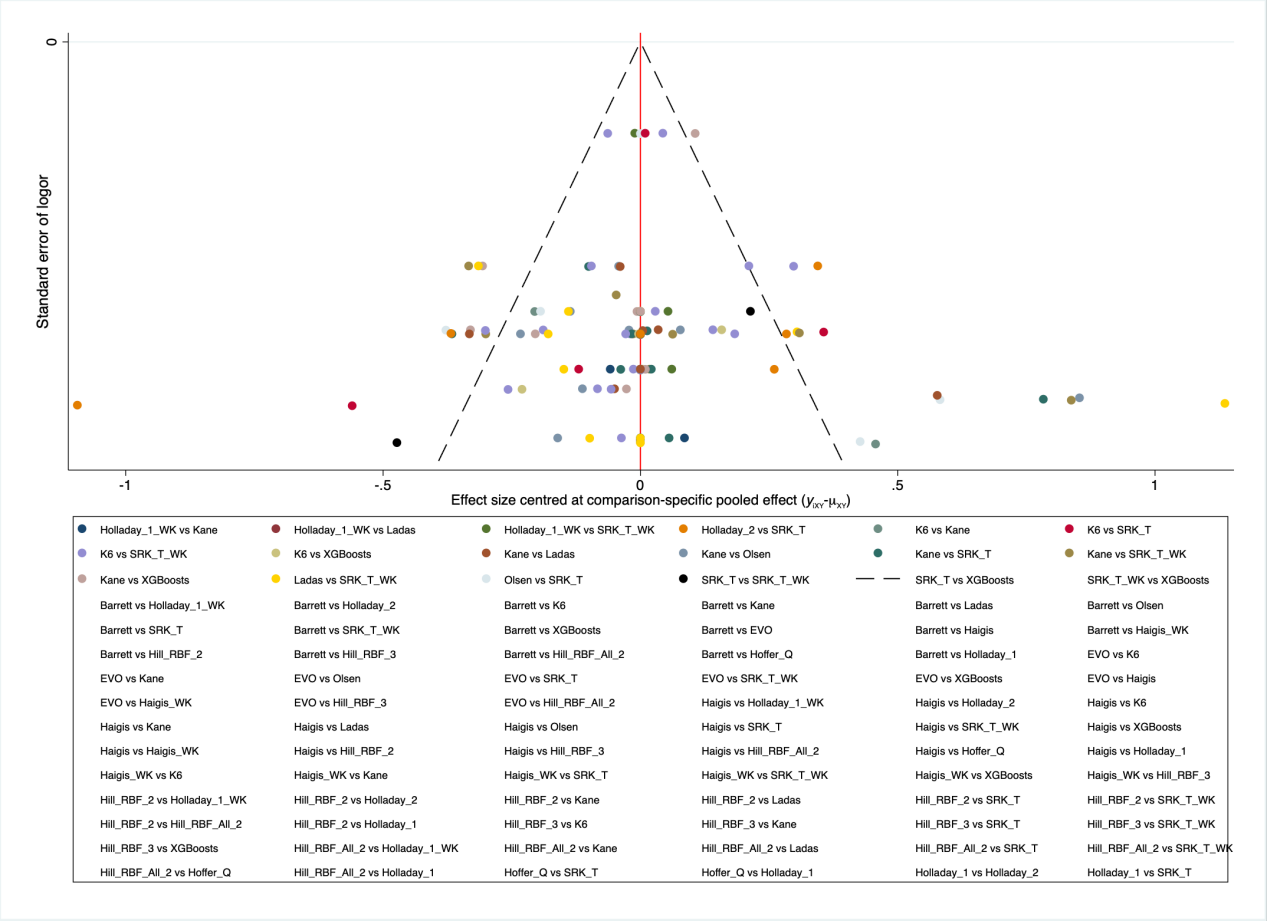


**Figure S3. The funnel plot of publication bias in mean absolute error (MAE).**


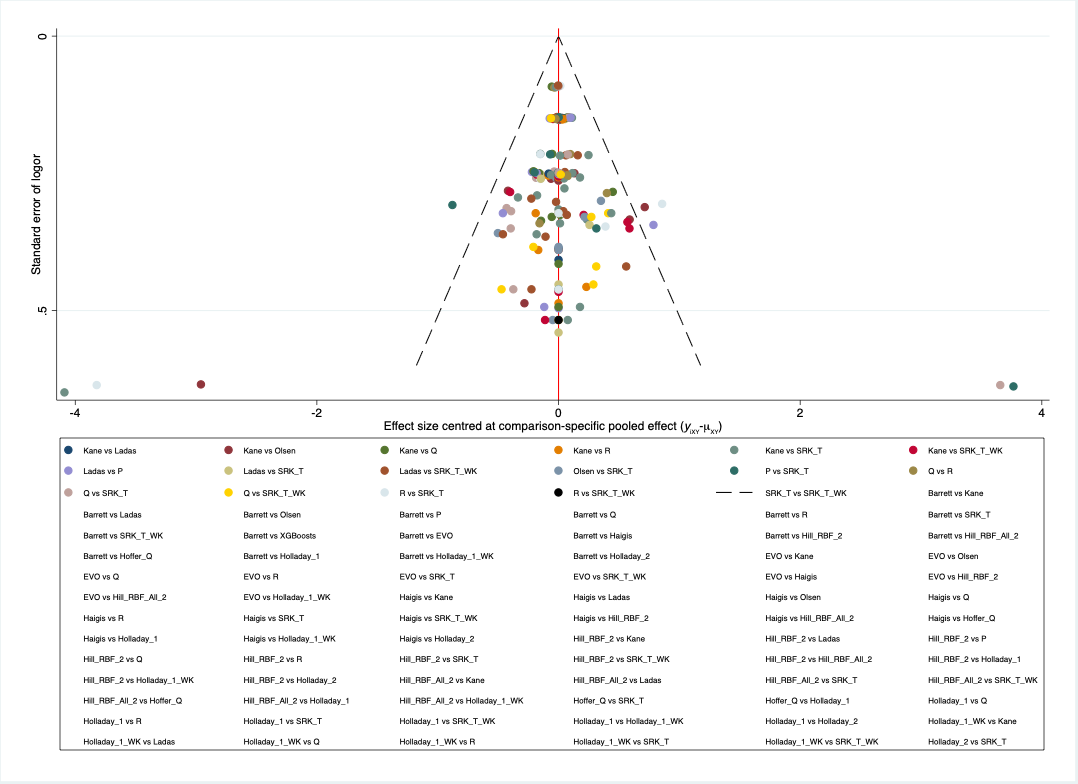


**Figure S4. The funnel plot of publication bias in percentage of eyes with prediction error within ± 0.25 D.**


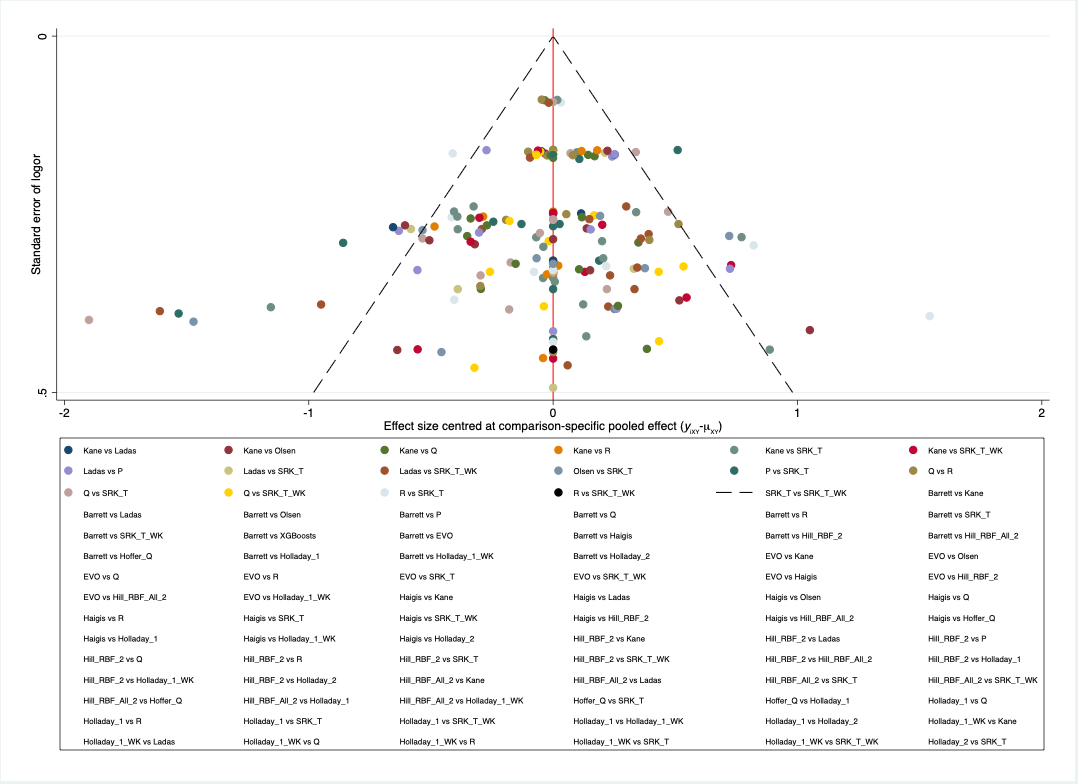


**Figure S5. The funnel plot of publication bias in percentage of eyes with prediction error within ± 0.50 D.**


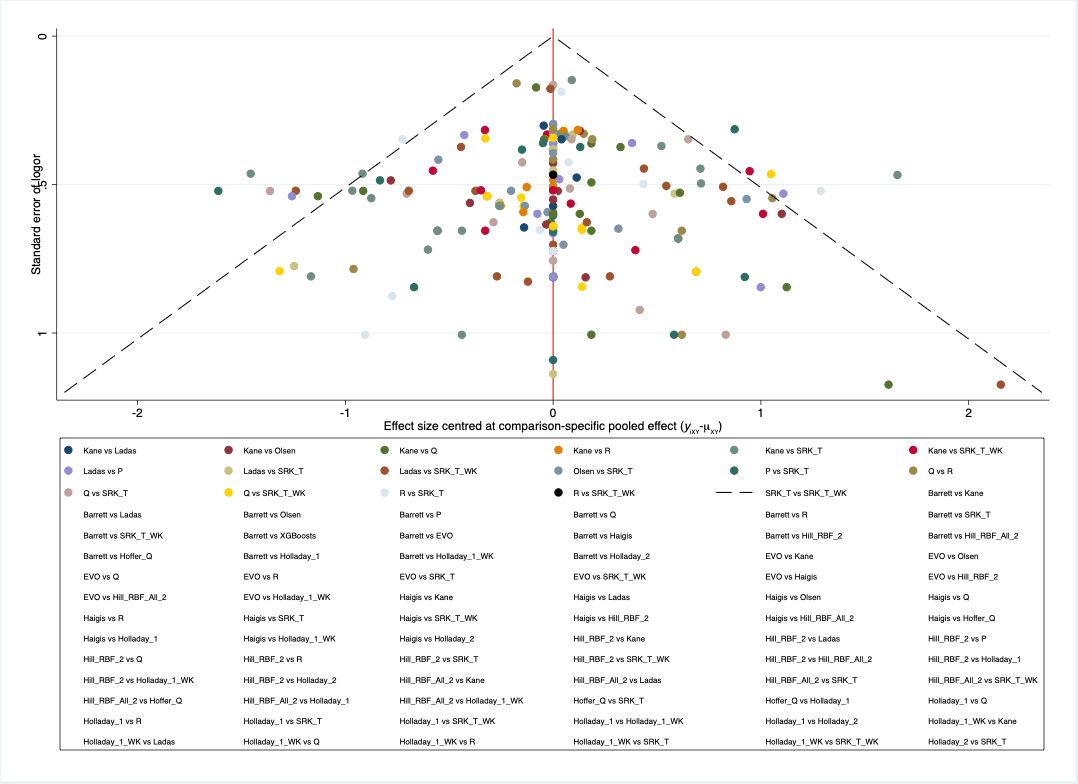


**Figure S6. The funnel plot of publication bias in percentage of eyes with prediction error within ± 1.00 D.**

**MEDLINE (OVID)**(“Myopia” OR “Long axial length*” OR Long AL OR Long eye*) AND ("Lenses, Intraocular"[Mesh] OR IOL OR intraocular lens) AND ( “Calculat*” OR “Formula*” OR “Prediction”)
---------------------------------------

**EMBASE**

1. ‘Myopia’
2. long axial length*
3. long AL*
4. long eye*
5. 1 or 2 or 3 or 4
6. ‘Lenses, Intraocular’
7. IOL
8. intraocular lens
9. 6 or 7 or 8
10. calculat*
11. formula*
12. prediction
13. 10 or 11 or 12
14. 5 and 9 and 13

---------------------------------------

**Cochrane Central Register of Controlled Trials (CENTRAL) in The Cochrane Library (Wiley)**#1 “Myopia” OR long axial length* OR long AL* OR long eye*

#2 “lens, intraocular” OR IOL OR intraocular lens*

#3 calculat* OR formula* OR “prediction”

#4 #1 AND #2 AND #3

---------------------------------------

**Web of Science**#1 (((TS=(Myopi*)) OR TS=(Long AL)) OR TS=(long axial length*)) OR TS=(long eye*)

#2 ((TS=(lens, intraocular)) OR TS=(IOL)) OR TS=(intraocular lens*)

#3 ((TS=(calculat*)) OR TS=(formula*)) OR TS=(prediction)

#4 #1 AND #2 AND #3

**Table S1. Literature search strategy.**

| **Study (Author, Year)** | **Country** | **Age (Mean ± SD, y)** | **Female** | **Total** | **Axial Lenth (mm)** | **Follow-up** | **Formulas** | **No. eyes** | **Percentage of IOL prediction error** | | | **MAE (D)** | **SD of MAE (D)** | **MedAE (D)** |
| --- | --- | --- | --- | --- | --- | --- | --- | --- | --- | --- | --- | --- | --- | --- |
|  |  |  |  |  |  |  |  |  | **Within ± 0.25 D** | **Within ± 0.50 D** | **Within ± 1.00 D** |  |  |  |
| Bernardes 2021 [17] | Portugal | 57.6 ± 8.7 | 36 | 46 | 30.89 ± 1.85 | 8 weeks | Barrett | 82 | 34.6 | 63 | 95.1 | 0.43 | 0.52 | 0.36 |
|  |  |  |  |  |  |  | Ladas | 82 | 15.2 | 38 | 81 | 0.66 | 0.75 | 0.61 |
|  |  |  |  |  |  |  | Hill-RBF 2 All | 82 | 43.1 | 70.6 | 94.1 | 0.42 | 0.51 | 0.37 |
|  |  |  |  |  |  |  | Hill-RBF 2 | 82 | 43.1 | 70.6 | 94.1 | 0.39 | 0.51 | 0.31 |
|  |  |  |  |  |  |  | Kane | 82 | 38.2 | 67.1 | 93.4 | 0.42 | 0.53 | 0.33 |
|  |  |  |  |  |  |  | SRK/T_WK | 82 | 34.6 | 66.7 | 91.4 | 0.47 | 0.6 | 0.37 |
|  |  |  |  |  |  |  | Haigis | 82 | 23.5 | 46.9 | 77.8 | 0.53 | 1.02 | 0.54 |
|  |  |  |  |  |  |  | Holladay 1_WK | 82 | 34.6 | 59.3 | 81.5 | 0.64 | 0.96 | 0.43 |
| Wan 2019 [18] | Hong Kong, China | 65.8 ± 9.1 |  | 127 | 27.2 ± 1.59 | 3 months | Hill-RBF 2 | 121 | 59.84 | 86.61 | 96.85 | 0.2 | 0.4 | 0.2 |
|  |  |  |  |  |  |  | Barrett | 121 | 55.91 | 86.61 | 98.42 | 0.21 | 0.39 | 0.21 |
|  |  |  |  |  |  |  | SRK/T | 121 | 47.24 | 82.68 | 95.28 | 0.27 | 0.49 | 0.27 |
|  |  |  |  |  |  |  | Holladay 1 | 121 | 41.73 | 70.87 | 94.49 | 0.3 | 0.5 | 0.3 |
|  |  |  |  |  |  |  | Hoffer Q | 121 | 38.58 | 73.23 | 94.49 | 0.33 | 0.54 | 0.33 |
|  |  |  |  |  |  |  | Haigis | 121 | 47.24 | 83.46 | 97.64 | 0.28 | 0.44 | 0.28 |
| Wei 2020 [19] | China | 62.61 ± 7.90 | 80 | 140 | 29.87 ± 2.13 |  | XGBoost | 140 | 49.66 | 78.28 | 97.24 | 0.33 | 0.28 | 0.25 |
|  |  |  |  |  |  |  | Barrett | 140 | 29.66 | 60.34 | 93.45 | 0.45 | 0.31 | 0.42 |
| Kane 2016 [20] | Australia |  |  | 47 |  | 5 years | Barrett | 47 | 38.3 | 76.6 | 95.7 | 0.375 |  | 0.325 |
|  |  |  |  |  |  |  | SRK/T | 47 | 44.7 | 66 | 97.9 | 0.365 |  | 0.358 |
|  |  |  |  |  |  |  | Ladas | 47 | 25.5 | 55.3 | 93.6 | 0.503 |  | 0.435 |
|  |  |  |  |  |  |  | Hill-RBF 2 | 47 | 44.7 | 68.1 | 95.7 | 0.373 |  | 0.31 |
|  |  |  |  |  |  |  | FullMonte IOL | 47 | 21.3 | 46.8 | 87.2 | 0.576 |  | 0.511 |
| Lin 2021 [21] | China | 61.78±11.43 | 97 | 175 | 28.66±2.028 | 3 months | Barrett | 175 | 44.6 | 74.9 | 98.9 | 0.342 | 0.253 | 0.297 |
|  |  |  |  |  |  |  | EVO | 175 | 45.7 | 82.3 | 99.4 | 0.314 | 0.216 | 0.288 |
|  |  |  |  |  |  |  | Haigis | 175 | 42.9 | 74.9 | 99.4 | 0.336 | 0.247 | 0.283 |
|  |  |  |  |  |  |  | Kane | 175 | 46.9 | 78.9 | 98.9 | 0.318 | 0.227 | 0.271 |
|  |  |  |  |  |  |  | SRK/T | 175 | 39.4 | 69.7 | 96 | 0.398 | 0.301 | 0.345 |
| Mo 2021 [22] | China | 60.72±12.04 | 60 | 106 | 28.87±1.88 | 1 month | Kane | 106 | 47.17 | 70.75 | 92.45 | 0.38 | 0.32 | 0.28 |
|  |  |  |  |  |  |  | EVO | 106 | 40.57 | 67.92 | 95.28 | 0.4 | 0.34 | 0.34 |
|  |  |  |  |  |  |  | Olsen | 106 | 34.91 | 64.15 | 97.17 | 0.44 | 0.3 | 0.4 |
|  |  |  |  |  |  |  | Barrett | 106 | 33.96 | 59.44 | 92.45 | 0.45 | 0.35 | 0.4 |
|  |  |  |  |  |  |  | SRK/T | 106 | 26.42 | 54.72 | 86.79 | 0.54 | 0.43 | 0.46 |
|  |  |  |  |  |  |  | Haigis | 106 | 21.7 | 34.91 | 81.13 | 0.67 | 0.45 | 0.63 |
| Guo 2022 [23] | China | 58.86±10.95 | 49 | 73 | 31.17±1.43 | 4 months | Kane | 73 | 41.1 | 67.12 | 94.52 | 0.37 | 0.31 | 0.3 |
|  |  |  |  |  |  |  | EVO | 73 | 36.88 | 63.01 | 94.52 | 0.46 | 0.34 | 0.39 |
|  |  |  |  |  |  |  | Olsen | 73 | 54.21 | 78.08 | 94.52 | 0.36 | 0.3 | 0.32 |
|  |  |  |  |  |  |  | Barrett | 73 | 41.1 | 71.23 | 94.52 | 0.4 | 0.33 | 0.34 |
|  |  |  |  |  |  |  | SRK/T | 73 | 24.66 | 20.55 | 60.27 | 0.8 | 0.44 | 0.78 |
|  |  |  |  |  |  |  | Haigis | 73 | 95.9 | 17.81 | 61.64 | 0.91 | 0.4 | 0.94 |
| Chen 2021 [24] | China | 61.33±9.09 | 540 | 1054 | 29.48±2.12 | 1 month | Kane | 1054 | 35.1 | 63.38 | 92.31 | 0.46 | 0.38 | 0.37 |
|  |  |  |  |  |  |  | Hill-RBF 2 | 1054 | 40.8 | 71.44 | 94.59 | 0.4 | 0.39 | 0.3 |
|  |  |  |  |  |  |  | Barrett | 1054 | 33.1 | 61.67 | 94.02 | 0.44 | 0.3 | 0.39 |
|  |  |  |  |  |  |  | EVO | 1054 | 30.7 | 59.01 | 87.57 | 0.58 | 0.69 | 0.42 |
| Ji 2019 [25] | China | 62±9 |  | 56 | 29.1±1.98 | 4 weeks | Barrett | 56 | 28.57 | 57.14 | 87.5 | 0.53 | 0.54 | 0.46 |
|  |  |  |  |  |  |  | Hill-RBF 2 | 56 | 19.64 | 51.79 | 83.93 | 0.58 | 0.56 | 0.47 |
|  |  |  |  |  |  |  | SRK/T | 56 | 17.86 | 46.43 | 85.73 | 0.59 | 0.56 | 0.53 |
|  |  |  |  |  |  |  | Haigis | 56 | 17.86 | 39.29 | 76.79 | 0.64 | 0.54 | 0.58 |
|  |  |  |  |  |  |  | Holladay 2 | 56 | 14.29 | 17.86 | 55.36 | 0.94 | 0.62 | 0.9 |
|  |  |  |  |  |  |  | Holladay 1 | 56 | 14.29 | 19.64 | 42.86 | 1.02 | 0.6 | 1.1 |
| Wei 2022 [26] | China | 64.99±9.33 | 52 | 103 | 28.85±2.34 | 2.43 ± 0.94 months | XGBoost | 103 |  |  |  | 0.409 | 0.398 | 0.296 |
|  |  |  |  |  |  |  | Hill-RBF 3 | 103 |  |  |  | 0.419 | 0.352 | 0.355 |
|  |  |  |  |  |  |  | Kane | 103 |  |  |  | 0.527 | 0.381 | 0.475 |
|  |  |  |  |  |  |  | Barrett | 103 |  |  |  | 0.536 | 0.414 | 0.445 |
|  |  |  |  |  |  |  | EVO | 103 |  |  |  | 0.532 | 0.412 | 0.425 |
|  |  |  |  |  |  |  | K6 | 103 |  |  |  | 0.572 | 0.487 | 0.415 |
|  |  |  |  |  |  |  | Haigis WK | 103 |  |  |  | 0.563 | 0.471 | 0.465 |
|  |  |  |  |  |  |  | Haigis | 103 |  |  |  | 0.584 | 0.483 | 0.475 |
|  |  |  |  |  |  |  | SRK/T_WK | 103 |  |  |  | 0.544 | 0.409 | 0.465 |
|  |  |  |  |  |  |  | SRK/T | 103 |  |  |  | 0.55 | 0.466 | 0.445 |
| Cheng 2020 [27] | China | 59.3±12.6 |  | 370 | 28.98±2.23 | 1 month | Kane | 370 | 47.62 | 74.80 | 95.83 | 0.34 |  | 0.27 |
|  |  |  |  |  |  |  | Hill-RBF 2 | 370 | 41.07 | 69.64 | 95.04 | 0.46 |  | 0.38 |
|  |  |  |  |  |  |  | Holladay 1_MWK | 370 | 43.65 | 76.39 | 95.83 | 0.35 |  | 0.27 |
|  |  |  |  |  |  |  | Holladay 1_WK | 370 | 45.83 | 76.19 | 96.43 | 0.39 |  | 0.32 |
|  |  |  |  |  |  |  | SRK/T_MWK | 370 | 35.91 | 71.23 | 93.85 | 0.41 |  | 0.33 |
|  |  |  |  |  |  |  | SRK/T_WK | 370 | 37.70 | 70.44 | 93.65 | 0.46 |  | 0.34 |
|  |  |  |  |  |  |  | EVO | 370 | 39.68 | 69.25 | 93.25 | 0.41 |  | 0.32 |
|  |  |  |  |  |  |  | Barrett | 370 | 42.26 | 72.82 | 95.83 | 0.39 |  | 0.33 |
|  |  |  |  |  |  |  | Haigis | 370 | 39.88 | 70.24 | 95.04 | 0.62 |  | 0.58 |
| Liu 2018 [28] | China | 61±11 |  | 92 | 28.85±2.02 | 1-3 months | Barrett | 136 | 44 | 78 | 97 | 0.32 |  | 0.27 |
|  |  |  |  |  |  |  | Hill-RBF 2 | 136 | 38 | 76 | 97 | 0.37 |  | 0.33 |
|  |  |  |  |  |  |  | Haigis | 136 | 39 | 68 | 95 | 0.41 |  | 0.36 |
|  |  |  |  |  |  |  | Holladay 1 | 136 | 35 | 58 | 95 | 0.45 |  | 0.4 |
|  |  |  |  |  |  |  | SRK/T | 136 | 38 | 60 | 87 | 0.49 |  | 0.34 |
|  |  |  |  |  |  |  | Holladay 1_WK | 136 | 40 | 72 | 99 | 0.37 |  | 0.34 |
|  |  |  |  |  |  |  | SRK/T_WK | 136 | 34 | 63 | 94 | 0.46 |  | 0.4 |
|  |  |  |  |  |  |  | Holladay 1_MWK | 136 | 38 | 64 | 97 | 0.39 |  | 0.38 |
|  |  |  |  |  |  |  | SRK/T_MWK | 136 | 33 | 60 | 93 | 0.47 |  | 0.43 |

**Table S2. Summary of studies included in the network meta-analysis**

| **Formula** | **No. Studies** | **No. Eyes** |
| --- | --- | --- |
| Kane | 7 | 2463 |
| Ladas | 2 | 129 |
| Hill-RBF 2 | 7 | 1866 |
| Hill-RBF 3 | 1 | 103 |
| Hill-RBF 2 All | 1 | 82 |
| XGBoosts | 2 | 243 |
| K6 | 1 | 103 |
| FullMonte IOL | 1 | 47 |
| Olsen | 2 | 179 |
| Barrett | 12 | 2463 |
| SRK/T | 8 | 817 |
| SRK/T_WK | 4 | 691 |
| SRK/T_MWK | 2 | 506 |
| Holladay 1 | 3 | 313 |
| Holladay 1_WK | 3 | 588 |
| Holladay 1_MWK | 2 | 506 |
| Holladay 2 | 1 | 56 |
| Hoffer Q | 1 | 121 |
| Haigis | 9 | 1222 |
| Haigis_WK | 1 | 103 |
| EVO | 6 | 1881 |

**Table S3. The number of trials and eyes involved in each formula.**

|  | **Bernardes 2021** | **Wan 2019** | **Wei 2020** | **Kane 2016** | **Lin 2021** | **Mo 2021** | **Guo 2022** | **Chen 2021** | **Ji 2019** | **Wei 2022** | **Cheng 2020** | **Liu 2018** |
| --- | --- | --- | --- | --- | --- | --- | --- | --- | --- | --- | --- | --- |
| Study objective |  |  |  |  |  |  |  |  |  |  |  |  |
| 1. Is the hypothesis/aim/objective of the study stated clearly in the abstract, introduction, or methods section? | Y | Y | Y | Y | Y | Y | Y | Y | Y | Y | Y | Y |
| Study population |  |  |  |  |  |  |  |  |  |  |  |  |
| 2. Are the characteristics of the participants included in the study described? | Y | Y | Y | Y | Y | Y | Y | Y | Y | Y | Y | Y |
| 3. Were the cases collected in more than on center? | N | N | Y | Y | Y | N | U | N | N | N | N | N |
| 4. Are the eligibility criteria (inclusion and exclusion criteria) for entry into the study explicit and appropriate? | Y | Y | Y | Y | Y | Y | Y | Y | Y | Y | Y | Y |
| 5. Were participants recruited consecutively? | Y | P | U | U | U | U | U | U | U | U | U | U |
| 6. Did participants enter the study at a similar point in the disease? | Y | Y | Y | Y | Y | Y | Y | Y | Y | Y | Y | Y |
| Intervention and co-intervention |  |  |  |  |  |  |  |  |  |  |  |  |
| 7. Was the intervention clearly described in the study? | Y | Y | Y | Y | P | Y | Y | Y | Y | Y | Y | Y |
| 8. Were additional interventions (co-interventions) clearly reported in the study? | Y | Y | Y | Y | U | Y | Y | Y | Y | Y | Y | Y |
| Outcome measure |  |  |  |  |  |  |  |  |  |  |  |  |
| 9. Are the outcome measures clearly defined in the introduction or methods section? | Y | Y | Y | Y | Y | Y | Y | Y | Y | Y | Y | Y |
| 10. Were relevant outcomes appropriately measured with objective and/or subjective methods? | Y | Y | Y | Y | Y | Y | Y | Y | Y | Y | Y | Y |
| 11. Were outcomes measured before and after intervention? | Y | Y | Y | Y | Y | Y | Y | Y | Y | Y | Y | Y |
| Statistical analysis |  |  |  |  |  |  |  |  |  |  |  |  |
| 12. Were the statistical tests used to assess the relevant outcomes appropriate? | Y | Y | Y | Y | Y | Y | Y | Y | Y | Y | Y | Y |
| Results and conclusions |  |  |  |  |  |  |  |  |  |  |  |  |
| 13. Was the length of follow-up reported? | Y | Y | Y | N | Y | Y | N | Y | N | Y | Y | Y |
| 14. Was the loss to the follow-up reported? | N | N | N | N | N | N | N | N | N | N | N | N |
| 15. Does the study provide estimates of the random variability in the data analysis of relevant outcomes? | Y | Y | Y | N | Y | Y | Y | Y | Y | Y | N | N |
| 16. Are adverse events reported? | N | N | N | N | N | N | N | N | N | N | N | N |
| 17. Are the conclusions of the study supported by results? | Y | Y | Y | Y | Y | Y | Y | Y | Y | Y | Y | Y |
| Competing interests and sources of support |  |  |  |  |  |  |  |  |  |  |  |  |
| 18. Are both competing interests and sources of support for the study reported? | Y | Y | Y | Y | Y | Y | Y | Y | Y | Y | Y | Y |
| New item |  |  |  |  |  |  |  |  |  |  |  |  |
| 19. Is this a prospective study? | N | N | N | N | N | N | N | N | N | N | N | N |
| 20. Was there the blinding of outcome assessment? | N | N | N | N | N | N | N | N | N | N | N | N |

**Table S4. Risk of bias within the included studies.**

| **Formulas** | **No. of studies** | **Range** |
| --- | --- | --- |
| Barrett | 12 | 0.21 - 0.46 |
| EVO | 6 | 0.288 - 0.425 |
| Full_Monte_IOL | 1 | 0.511 |
| Haigis | 9 | 0.28 - 0.94 |
| Haigis_WK | 1 | 0.465 |
| Hill_RBF_2 | 7 | 0.2 - 0.47 |
| Hill_RBF_3 | 1 | 0.355 |
| Hill_RBF_All_2 | 1 | 0.37 |
| Hoffer_Q | 1 | 0.33 |
| Holladay_1 | 3 | 0.3 - 1.1 |
| Holladay_1_MWK | 2 | 0.27 - 0.38 |
| Holladay_1_WK | 3 | 0.32 - 0.43 |
| Holladay_2 | 1 | 0.9 |
| K6 | 1 | 0.415 |
| Kane | 7 | 0.27 - 0.475 |
| Ladas | 2 | 0.435 - 0.61 |
| Olsen | 2 | 0.32 - 0.4 |
| SRK_T | 8 | 0.27 - 0.78 |
| SRK_T_MWK | 2 | 0.33 - 0.43 |
| SRK_T_WK | 4 | 0.43 - 0.465 |
| XGBoost | 2 | 0.25 - 0.296 |

**Table S5. A descriptive analysis for median absolute error.**

| **Formulas** | **Sucra** | **PrBest** |
| --- | --- | --- |
| Barrett | 0.663729412 | 0.00125 |
| EVO | 0.623808824 | 0.00415 |
| Haigis | 0.284664706 | 0 |
| Haigis_WK | 0.503644118 | 0.02395 |
| Hill_RBF_2 | 0.701129412 | 0.025 |
| Hill_RBF_3 | 0.822702941 | 0.2604 |
| Hill_RBF_All_2 | 0.645264706 | 0.1063 |
| Hoffer_Q | 0.270829412 | 0.00325 |
| Holladay_1 | 0.124332353 | 0 |
| Holladay_1_WK | 0.237714706 | 0.00655 |
| Holladay_2 | 0.062079412 | 0.00045 |
| K6 | 0.483688235 | 0.01845 |
| Kane | 0.751964706 | 0.01675 |
| Ladas | 0.198002941 | 0.002 |
| Olsen | 0.846 | 0.21345 |
| SRK_T | 0.3656 | 0 |
| SRK_T_WK | 0.540661765 | 0.00925 |
| XGBoost | 0.874182353 | 0.3088 |

**Table S6. Results of network rank test in mean absolute error (MAE).**

| **Formulas** | **Sucra** | **PrBest** |
| --- | --- | --- |
| Barrett | 0.594551471 | 0.000375 |
| EVO | 0.499375 | 0.001125 |
| Full_Monte_IOL | 0.089029412 | 0.001 |
| Haigis | 0.642463235 | 0.004875 |
| Hill_RBF_2 | 0.709198529 | 0.00925 |
| Hill_RBF_All_2 | 0.808860294 | 0.2145 |
| Hoffer_Q | 0.230632353 | 0.00325 |
| Holladay_1 | 0.316514706 | 0.001125 |
| Holladay_1_MWK | 0.593154412 | 0.018 |
| Holladay_1_WK | 0.6585 | 0.015375 |
| Holladay_2 | 0.327448529 | 0.01825 |
| Kane | 0.719463235 | 0.01475 |
| Ladas | 0.074264706 | 0.000125 |
| Olsen | 0.648823529 | 0.04025 |
| SRK_T | 0.319977941 | 0 |
| SRK_T_MWK | 0.372926471 | 0.0015 |
| SRK_T_WK | 0.473404412 | 0.00375 |
| XGBoost | 0.921411765 | 0.6525 |

**Table S7. Results of network rank test in percentage of eyes within ±0.25 D of the prediction error.**

| **Formulas** | **Sucra** | **PrBest** |
| --- | --- | --- |
| Barrett | 0.700573529 | 0.00025 |
| EVO | 0.70725 | 0.002 |
| Full_Monte_IOL | 0.120176471 | 0 |
| Haigis | 0.317492647 | 0 |
| Hill_RBF_2 | 0.693588235 | 0.001875 |
| Hill_RBF_All_2 | 0.779 | 0.092 |
| Hoffer_Q | 0.193514706 | 0 |
| Holladay_1 | 0.145713235 | 0 |
| Holladay_1_MWK | 0.564830882 | 0.00325 |
| Holladay_1_WK | 0.618941176 | 0.001625 |
| Holladay_2 | 0.048419118 | 0 |
| Kane | 0.781970588 | 0.00475 |
| Ladas | 0.171147059 | 0 |
| Olsen | 0.9195 | 0.2445 |
| SRK_T | 0.345838235 | 0 |
| SRK_T_MWK | 0.426463235 | 0.00025 |
| SRK_T_WK | 0.517786765 | 0.00025 |
| XGBoost | 0.947794118 | 0.64925 |

**Table S8. Results of network rank test in percentage of eyes within ±0.50D of the prediction error.**

| **Formulas** | **Sucra** | **PrBest** |
| --- | --- | --- |
| Barrett | 0.594551471 | 0.000375 |
| EVO | 0.499375 | 0.001125 |
| Full_Monte_IOL | 0.089029412 | 0.001 |
| Haigis | 0.642463235 | 0.004875 |
| Hill_RBF_2 | 0.709198529 | 0.00925 |
| Hill_RBF_All_2 | 0.808860294 | 0.2145 |
| Hoffer_Q | 0.230632353 | 0.00325 |
| Holladay_1 | 0.316514706 | 0.001125 |
| Holladay_1_MWK | 0.593154412 | 0.018 |
| Holladay_1_WK | 0.6585 | 0.015375 |
| Holladay_2 | 0.327448529 | 0.01825 |
| Kane | 0.719463235 | 0.01475 |
| Ladas | 0.074264706 | 0.000125 |
| Olsen | 0.648823529 | 0.04025 |
| SRK_T | 0.319977941 | 0 |
| SRK_T_MWK | 0.372926471 | 0.0015 |
| SRK_T_WK | 0.473404412 | 0.00375 |
| XGBoost | 0.921411765 | 0.6525 |

**Table S9. Results of network rank test in percentage of eyes within ±1.00D of the prediction error.**

| **Comparisons** | **Direct estimate (95%)** | | **Indirect estimate (95%)** | | **Network (95%)** | | **p - value** |
| --- | --- | --- | --- | --- | --- | --- | --- |
|  | **Coefficient** | **CrI** | **Coefficient** | **CrI** | **Coefficient** | **CrI** |  |
| EVO.Haigis | 0.19 | (0.058, 0.33) | -0.038 | (-0.30, 0.22) | 0.14 | (0.021, 0.26) | 0.113575 |
| EVO.Hill_RBF_2 | -0.18 | (-0.44, 0.083) | 0.043 | (-0.13, 0.22) | -0.027 | (-0.17, 0.12) | 0.1554 |
| dEVO.SRK_T | 0.14 | (-0.0038, 0.29) | -0.064 | (-0.36, 0.23) | 0.1 | (-0.019, 0.23) | 0.20705 |
| EVO.SRK_T_WK | 0.014 | (-0.28, 0.30) | 0.043 | (-0.24, 0.33) | 0.035 | (-0.15, 0.22) | 0.8797 |
| EVO.XGBoost | -0.12 | (-0.42, 0.17) | -0.13 | (-0.43, 0.18) | -0.11 | (-0.30, 0.074) | 0.99055 |
| Haigis.Hill_RBF_2 | -0.088 | (-0.28, 0.10) | -0.28 | (-0.54, -0.032) | -0.17 | (-0.30, -0.029) | 0.21 |
| Haigis.Kane | -0.2 | (-0.35, -0.062) | -0.11 | (-0.42, 0.20) | -0.18 | (-0.30, -0.068) | 0.550125 |
| Haigis.XGBoost | -0.18 | (-0.47, 0.11) | -0.29 | (-0.59, 0.0066) | -0.25 | (-0.44, -0.070) | 0.569525 |
| Hill_RBF_2.Kane | 0.048 | (-0.16, 0.25) | -0.099 | (-0.31, 0.11) | -0.015 | (-0.16, 0.12) | 0.309425 |
| Hill_RBF_2.SRK_T | 0.045 | (-0.17, 0.26) | 0.21 | (0.0068, 0.40) | 0.13 | (-0.013, 0.27) | 0.270425 |
| Hill_RBF_2.SRK_T_WK | 0.08 | (-0.24, 0.40) | 0.051 | (-0.21, 0.31) | 0.061 | (-0.13, 0.25) | 0.885975 |
| Kane.SRK_T | 0.17 | (0.016, 0.32) | 0.073 | (-0.19, 0.34) | 0.15 | (0.027, 0.26) | 0.521875 |
| Kane.XGBoost | -0.12 | (-0.40, 0.17) | -0.08 | (-0.38, 0.22) | -0.071 | (-0.26, 0.11) | 0.8486 |
| SRK_T.SRK_T_WK | -0.0056 | (-0.29, 0.28) | -0.081 | (-0.36, 0.20) | -0.068 | (-0.25, 0.11) | 0.703125 |
| SRK_T.XGBoost | -0.14 | (-0.43, 0.15) | -0.25 | (-0.55, 0.048) | -0.22 | (-0.40, -0.029) | 0.57825 |
| SRK_T_WK.XGBoost | -0.14 | (-0.43, 0.16) | -0.17 | (-0.56, 0.21) | -0.15 | (-0.37, 0.073) | 0.870325 |

**Table S10. Node-splitting analysis of inconsistency in mean absolute error (MAE).**

| **Comparisons** | **Direct estimate (95%)** | | **Indirect estimate (95%)** | | **Network (95%)** | | **p - value** |
| --- | --- | --- | --- | --- | --- | --- | --- |
|  | **Coefficient** | **CrI** | **Coefficient** | **CrI** | **Coefficient** | **CrI** |  |
| EVO.Haigis | 0.36 | (-0.16, 0.94) | -0.13 | (-1.1, 0.78) | 0.14 | (-0.34, 0.64) | 0.33375 |
| EVO.Hill_RBF_2 | 0.26 | (-0.58, 1.1) | 0.18 | (-0.59, 0.95) | 0.22 | (-0.30, 0.74) | 0.884775 |
| EVO.Holladay_1_MWK | 0.17 | (-0.98, 1.3) | 0.014 | (-1.0, 1.1) | 0.1 | (-0.61, 0.81) | 0.841475 |
| EVO.Holladay_1_WK | 0.26 | (-0.90, 1.4) | 0.12 | (-0.72, 0.96) | 0.17 | (-0.46, 0.82) | 0.8447 |
| EVO.SRK_T | -0.46 | (-1.1, 0.18) | 0.069 | (-0.66, 0.81) | -0.21 | (-0.72, 0.31) | 0.26445 |
| EVO.SRK_T_MWK | -0.16 | (-1.3, 0.99) | -0.21 | (-1.3, 0.86) | -0.18 | (-0.89, 0.53) | 0.952525 |
| EVO.SRK_T_WK | -0.078 | (-1.2, 1.1) | 0.0037 | (-0.85, 0.85) | -0.036 | (-0.67, 0.61) | 0.904375 |
| Haigis.Hill_RBF_2 | 0.27 | (-0.29, 0.85) | -0.34 | (-1.3, 0.57) | 0.076 | (-0.39, 0.52) | 0.2485 |
| Haigis.Kane | 0.0039 | (-0.53, 0.49) | 0.21 | (-0.77, 1.2) | 0.091 | (-0.39, 0.54) | 0.69745 |
| Haigis.Ladas | -0.58 | (-1.9, 0.74) | -1.1 | (-2.3, 0.091) | -1.1 | (-1.9, -0.28) | 0.559725 |
| Haigis.SRK_T | -0.52 | (-1.0, -0.050) | 0.43 | (-0.81, 1.7) | -0.35 | (-0.81, 0.097) | 0.150425 |
| Hill_RBF_2.Kane | -0.052 | (-0.76, 0.66) | 0.093 | (-0.73, 0.90) | 0.014 | (-0.48, 0.50) | 0.784175 |
| Hill_RBF_2.SRK_T | -0.15 | (-0.81, 0.53) | -0.86 | (-1.7, -0.055) | -0.43 | (-0.91, 0.053) | 0.169275 |
| Holladay_1.Holladay_1_MWK | 0.13 | (-1.1, 1.3) | 0.56 | (-0.59, 1.7) | 0.36 | (-0.44, 1.2) | 0.594125 |
| Holladay_1.Holladay_1_WK | 0.22 | (-0.99, 1.4) | 0.6 | (-0.41, 1.6) | 0.43 | (-0.30, 1.2) | 0.62335 |
| Holladay_1.SRK_T_MWK | -0.1 | (-1.3, 1.1) | 0.22 | (-0.93, 1.4) | 0.073 | (-0.72, 0.88) | 0.69135 |
| Holladay_1.SRK_T_WK | -0.033 | (-1.2, 1.2) | 0.41 | (-0.59, 1.4) | 0.22 | (-0.50, 0.97) | 0.573175 |
| Holladay_1_MWK.Kane | 0.16 | (-0.98, 1.3) | 0.19 | (-0.83, 1.2) | 0.13 | (-0.57, 0.83) | 0.975725 |
| Holladay_1_MWK.SRK_T | -0.00084 | (-1.2, 1.2) | -0.46 | (-1.4, 0.51) | -0.31 | (-1.0, 0.38) | 0.5446 |
| Holladay_1_WK.Kane | 0.11 | (-0.77, 1.0) | 0.059 | (-1.0, 1.1) | 0.056 | (-0.56, 0.67) | 0.935125 |
| Holladay_1_WK.Ladas | -1.1 | (-2.5, 0.21) | -1.1 | (-2.4, 0.29) | -1.1 | (-2.0, -0.23) | 0.938 |
| Holladay_1_WK.SRK_T | -0.092 | (-1.3, 1.1) | -0.5 | (-1.3, 0.28) | -0.39 | (-1.0, 0.23) | 0.5655 |
| Kane.Ladas | -1.3 | (-2.6, 0.058) | -1.1 | (-2.4, 0.16) | -1.2 | (-2.0, -0.34) | 0.82535 |
| Kane.SRK_T | -0.65 | (-1.3, 0.0028) | -0.22 | (-0.89, 0.46) | -0.44 | (-0.93, 0.053) | 0.34895 |
| Kane.SRK_T_MWK | -0.49 | (-1.6, 0.65) | -0.41 | (-1.4, 0.61) | -0.41 | (-1.1, 0.29) | 0.9142 |
| Kane.SRK_T_WK | -0.3 | (-1.2, 0.57) | -0.32 | (-1.4, 0.76) | -0.27 | (-0.88, 0.35) | 0.977 |
| Ladas.SRK_T | 1 | (-0.40, 2.4) | 0.72 | (-0.40, 1.9) | 0.72 | (-0.094, 1.6) | 0.763875 |
| Ladas.SRK_T_WK | 1.1 | (-0.21, 2.5) | 0.75 | (-0.57, 2.1) | 0.9 | (0.018, 1.8) | 0.68545 |
| SRK_T.SRK_T_MWK | -0.23 | (-1.4, 0.98) | 0.13 | (-0.85, 1.1) | 0.03 | (-0.66, 0.73) | 0.64205 |
| SRK_T.SRK_T_WK | -0.16 | (-1.4, 1.1) | 0.3 | (-0.47, 1.1) | 0.17 | (-0.44, 0.80) | 0.50835 |

**Table S11. Node-splitting analysis of percentage of eyes within ± 0.25 D of the prediction error.**

| **Comparisons** | **Direct estimate (95%)** | | **Indirect estimate (95%)** | | **Network (95%)** | | **p - value** |
| --- | --- | --- | --- | --- | --- | --- | --- |
|  | **Coefficient** | **CrI** | **Coefficient** | **CrI** | **Coefficient** | **CrI** |  |
| EVO.Haigis | 0.36 | (-0.16, 0.94) | -0.13 | (-1.1, 0.79) | 0.14 | (-0.34, 0.64) | 0.33295 |
| EVO.Hill_RBF_2 | 0.26 | (-0.58, 1.1) | 0.18 | (-0.58, 0.94) | 0.22 | (-0.31, 0.73) | 0.881875 |
| EVO.Holladay_1_MWK | 0.17 | (-0.99, 1.3) | 0.016 | (-1.0, 1.1) | 0.1 | (-0.61, 0.82) | 0.84045 |
| EVO.Holladay_1_WK | 0.26 | (-0.90, 1.4) | 0.12 | (-0.71, 0.97) | 0.18 | (-0.46, 0.83) | 0.843225 |
| EVO.SRK_T | -0.46 | (-1.1, 0.18) | 0.069 | (-0.66, 0.81) | -0.21 | (-0.73, 0.31) | 0.95005 |
| EVO.SRK_T_MWK | -0.16 | (-1.3, 0.99) | -0.21 | (-1.3, 0.85) | -0.18 | (-0.90, 0.55) | 0.9077 |
| EVO.SRK_T_WK | -0.082 | (-1.2, 1.1) | -0.0028 | (-0.84, 0.85) | -0.037 | (-0.67, 0.62) | 0.25005 |
| Haigis.Hill_RBF_2 | 0.27 | (-0.30, 0.85) | -0.34 | (-1.3, 0.57) | 0.075 | (-0.39, 0.53) | 0.25005 |
| Haigis.Kane | 0.0023 | (-0.53, 0.49) | 0.22 | (-0.77, 1.2) | 0.093 | (-0.38, 0.55) | 0.690125 |
| Haigis.Ladas | -0.58 | (-1.9, 0.75) | -1.1 | (-2.3, 0.11) | -1.1 | (-1.9, -0.25) | 0.566475 |
| Haigis.SRK_T | -0.52 | (-1.0, -0.049) | 0.43 | (-0.80, 1.7) | -0.35 | (-0.82, 0.097) | 0.15595 |
| Hill_RBF_2.Kane | -0.054 | (-0.77, 0.66) | 0.084 | (-0.74, 0.89) | 0.017 | (-0.48, 0.51) | 0.79035 |
| Hill_RBF_2.SRK_T | -0.15 | (-0.81, 0.52) | -0.85 | (-1.7, -0.052) | -0.43 | (-0.91, 0.057) | 0.17405 |
| Holladay_1.Holladay_1_MWK | 0.13 | (-1.1, 1.3) | 0.57 | (-0.59, 1.7) | 0.36 | (-0.45, 1.2) | 0.59445 |
| Holladay_1.Holladay_1_WK | 0.22 | (-0.98, 1.4) | 0.6 | (-0.41, 1.6) | 0.43 | (-0.31, 1.2) | 0.6231 |
| Holladay_1.SRK_T_MWK | -0.098 | (-1.3, 1.1) | 0.23 | (-0.93, 1.4) | 0.078 | (-0.73, 0.88) | 0.6912 |
| Holladay_1.SRK_T_WK | -0.035 | (-1.3, 1.2) | 0.41 | (-0.60, 1.4) | 0.22 | (-0.52, 0.97) | 0.572175 |
| Holladay_1_MWK.Kane | 0.17 | (-0.98, 1.3) | 0.19 | (-0.84, 1.2) | 0.13 | (-0.57, 0.83) | 0.97845 |
| Holladay_1_MWK.SRK_T | -0.0029 | (-1.2, 1.2) | -0.46 | (-1.4, 0.50) | -0.32 | (-1.0, 0.38) | 0.5424 |
| Holladay_1_WK.Kane | 0.11 | (-0.76, 1.0) | 0.056 | (-1.0, 1.1) | 0.055 | (-0.56, 0.67) | 0.93315 |
| Holladay_1_WK.Ladas | -1.1 | (-2.5, 0.20) | -1.1 | (-2.4, 0.26) | -1.1 | (-2.0, -0.22) | 0.9336 |
| Holladay_1_WK.SRK_T | -0.093 | (-1.3, 1.1) | -0.49 | (-1.3, 0.29) | -0.39 | (-1.0, 0.23) | 0.570125 |
| Kane.Ladas | -1.3 | (-2.6, 0.045) | -1.1 | (-2.4, 0.16) | -1.2 | (-2.0, -0.33) | 0.834275 |
| Kane.SRK_T | -0.65 | (-1.3, 0.0015) | -0.22 | (-0.89, 0.46) | -0.44 | (-0.94, 0.051) | 0.35555 |
| Kane.SRK_T_MWK | -0.5 | (-1.6, 0.66) | -0.41 | (-1.4, 0.62) | -0.41 | (-1.1, 0.29) | 0.9108 |
| Kane.SRK_T_WK | -0.3 | (-1.2, 0.57) | -0.32 | (-1.4, 0.77) | -0.27 | (-0.88, 0.35) | 0.97635 |
| Ladas.SRK_T | 1 | (-0.40, 2.4) | 0.73 | (-0.39, 1.9) | 0.72 | (-0.11, 1.6) | 0.764875 |
| Ladas.SRK_T_WK | 1.1 | (-0.20, 2.5) | 0.77 | (-0.58, 2.1) | 0.9 | (0.0062, 1.8) | 0.699475 |
| SRK_T.SRK_T_MWK | -0.23 | (-1.5, 0.97) | 0.12 | (-0.84, 1.1) | 0.032 | (-0.67, 0.74) | 0.639875 |
| SRK_T.SRK_T_WK | -0.16 | (-1.4, 1.0) | 0.3 | (-0.47, 1.1) | 0.18 | (-0.44, 0.80) | 0.511875 |

**Table S12. Node-splitting analysis of percentage of eyes within ± 0.50 D of the prediction error.**

| **Comparisons** | **Direct estimate (95%)** | | **Indirect estimate (95%)** | | **Network (95%)** | | **p - value** |
| --- | --- | --- | --- | --- | --- | --- | --- |
|  | **Coefficient** | **CrI** | **Coefficient** | **CrI** | **Coefficient** | **CrI** |  |
| EVO.Haigis | -0.89 | (-1.8, -0.038) | 0.034 | (-1.3, 1.4) | -0.64 | (-1.3, 0.023) | 0.241325 |
| EVO.Hill_RBF_2 | 0.64 | (-0.27, 1.5) | -0.77 | (-1.8, 0.28) | 0.083 | (-0.66, 0.78) | 0.049 |
| EVO.Holladay_1_MWK | 0.48 | (-0.97, 2.0) | -0.2 | (-1.7, 1.4) | 0.047 | (-0.97, 1.1) | 0.527125 |
| EVO.Holladay_1_WK | 0.63 | (-0.85, 2.1) | -0.4 | (-1.6, 0.77) | -0.023 | (-0.93, 0.86) | 0.276775 |
| EVO.SRK_T | -1.6 | (-2.6, -0.66) | -0.047 | (-1.0, 0.95) | -0.83 | (-1.6, -0.14) | 0.0243 |
| EVO.SRK_T_MWK | 0.089 | (-1.3, 1.5) | -1 | (-2.4, 0.41) | -0.51 | (-1.5, 0.43) | 0.276675 |
| EVO.SRK_T_WK | 0.039 | (-1.4, 1.5) | -0.49 | (-1.6, 0.63) | -0.32 | (-1.2, 0.53) | 0.5624 |
| Haigis.Hill_RBF_2 | 0.45 | (-0.31, 1.2) | 1.4 | (0.037, 2.6) | 0.72 | (0.080, 1.3) | 0.230375 |
| Haigis.Kane | 1 | (0.27, 1.8) | 0.36 | (-1.1, 1.8) | 0.88 | (0.24, 1.5) | 0.39095 |
| Haigis.Ladas | 0.22 | (-1.3, 1.7) | -0.22 | (-2.0, 1.6) | -0.13 | (-1.2, 0.91) | 0.712675 |
| Haigis.SRK_T | -0.21 | (-0.77, 0.31) | 2.1 | (-0.14, 5.4) | -0.2 | (-0.80, 0.40) | 0.0486 |
| Hill_RBF_2.Kane | -0.14 | (-1.0, 0.75) | 0.68 | (-0.46, 1.8) | 0.16 | (-0.51, 0.85) | 0.26025 |
| Hill_RBF_2.SRK_T | -0.38 | (-1.3, 0.52) | -1.5 | (-2.5, -0.54) | -0.92 | (-1.6, -0.24) | 0.087375 |
| Holladay_1.Holladay_1_MWK | 0.4 | (-1.3, 2.1) | 1.9 | (0.49, 3.2) | 1.3 | (0.17, 2.4) | 0.186625 |
| Holladay_1.Holladay_1_WK | 1.5 | (-0.44, 3.8) | 1.5 | (0.35, 2.6) | 1.2 | (0.20, 2.2) | 0.9863 |
| Holladay_1.SRK_T_MWK | -0.4 | (-2.0, 1.2) | 1.5 | (0.13, 2.8) | 0.71 | (-0.35, 1.8) | 0.07315 |
| Holladay_1.SRK_T_WK | -0.28 | (-1.9, 1.3) | 1.6 | (0.44, 2.8) | 0.91 | (-0.080, 1.9) | 0.057625 |
| Holladay_1_MWK.Kane | -0.0013 | (-1.5, 1.5) | 0.29 | (-1.3, 1.8) | 0.19 | (-0.80, 1.2) | 0.78815 |
| Holladay_1_MWK.SRK_T | -1.5 | (-3.1, 0.18) | -0.73 | (-2.0, 0.51) | -0.88 | (-1.9, 0.095) | 0.480925 |
| Holladay_1_WK.Kane | 0.44 | (-0.65, 1.5) | -0.88 | (-3.1, 0.90) | 0.27 | (-0.60, 1.1) | 0.22075 |
| Holladay_1_WK.Ladas | 0.0092 | (-1.5, 1.5) | -1.3 | (-3.2, 0.64) | -0.75 | (-1.9, 0.39) | 0.2837 |
| Holladay_1_WK.SRK_T | -2.5 | (-4.9, -0.63) | -0.36 | (-1.3, 0.61) | -0.81 | (-1.7, 0.051) | 0.04635 |
| Kane.Ladas | -1.2 | (-2.9, 0.48) | -0.99 | (-2.8, 0.90) | -1 | (-2.1, 0.059) | 0.866 |
| Kane.SRK_T | -1.4 | (-2.4, -0.41) | -0.7 | (-1.7, 0.31) | -1.1 | (-1.8, -0.40) | 0.310425 |
| Kane.SRK_T_MWK | -0.39 | (-1.9, 1.1) | -1.1 | (-2.5, 0.31) | -0.75 | (-1.7, 0.18) | 0.4828 |
| Kane.SRK_T_WK | -0.39 | (-1.5, 0.73) | -0.96 | (-2.4, 0.50) | -0.56 | (-1.4, 0.26) | 0.532525 |
| Ladas.SRK_T | 1.8 | (-0.74, 5.3) | -0.22 | (-1.5, 1.0) | -0.068 | (-1.1, 0.98) | 0.167425 |
| Ladas.SRK_T_WK | 0.84 | (-0.75, 2.5) | 0.27 | (-1.7, 2.2) | 0.45 | (-0.69, 1.6) | 0.647275 |
| SRK_T.SRK_T_MWK | 0.67 | (-0.82, 2.2) | 0.36 | (-0.85, 1.6) | 0.32 | (-0.62, 1.2) | 0.7492 |
| SRK_T.SRK_T_WK | 0.79 | (-0.72, 2.3) | 0.5 | (-0.49, 1.5) | 0.52 | (-0.32, 1.3) | 0.74945 |

**Table S13. Node-splitting analysis of percentage of eyes within ± 1.00 D of the prediction error.**

| **Study (Author, Year)** | **Formulas** | **No. eyes** | **Percentage of IOL prediction error** | | | **MAE (D)** | **SD of MAE (D)** | **MedAE (D)** |
| --- | --- | --- | --- | --- | --- | --- | --- | --- |
|  |  |  | **Within ± 0.25 D** | **Within ± 0.50 D** | **Within ± 1.00 D** |  |  |  |
| Wei 2020 [19] | XGBoost | 23 | - | - | - | 0.35 | 0.26 | 0.3 |
|  | Barrett | 23 | - | - | - | 0.35 | 0.24 | 0.38 |
| Lin 2021 [21] | Barrett | 81 | 45.7 | 76.6 | 98.8 | - | - | - |
|  | EVO | 81 | 45.7 | 88.9 | 98.8 | - | - | - |
|  | Haigis | 81 | 46.9 | 76.5 | 98.8 | - | - | - |
|  | Kane | 81 | 45.7 | 79 | 98.8 | - | - | - |
|  | SRK/T | 81 | 44.4 | 69.1 | 93.8 | - | - | - |
| Chen 2021 [24] | Kane | 312 | - | - | - | 0.48 | 0.36 | 0.42 |
|  | Hill-RBF 2 | 312 | - | - | - | 0.39 | 0.34 | 0.3 |
|  | Barrett | 312 | - | - | - | 0.43 | 0.3 | 0.37 |
|  | EVO | 312 | - | - | - | 0.47 | 0.45 | 0.4 |
| Ji 2019 [25] | Barrett | 19 | - | - | - | 0.49 | - | - |
|  | Hill-RBF 2 | 19 | - | - | - | 0.41 | - | - |
|  | SRK/T | 19 | - | - | - | 0.18 | - | - |
|  | Haigis | 19 | - | - | - | 0.45 | - | - |
|  | Holladay 2 | 19 | - | - | - | 0.68 | - | - |
|  | Holladay 1 | 19 | - | - | - | 0.62 | - | - |
| Wei 2022 [26] | XGBoost | 46 | - | - | - | 0.418 | 0.33 | 0.409 |
|  | Hill-RBF 3 | 46 | - | - | - | 0.463 | 0.313 | 0.375 |
|  | Kane | 46 | - | - | - | 0.48 | 0.348 | 0.47 |
|  | Barrett | 46 | - | - | - | 0.549 | 0.374 | 0.47 |
|  | EVO | 46 | - | - | - | 0.593 | 0.403 | 0.535 |
|  | K6 | 46 | - | - | - | 0.651 | 0.493 | 0.613 |
|  | Haigis WK | 46 | - | - | - | 0.628 | 0.46 | 0.518 |
|  | Haigis | 46 | - | - | - | 0.501 | 0.377 | 0.405 |
|  | SRK/T_WK | 46 | - | - | - | 0.542 | 0.324 | 0.47 |
|  | SRK/T | 46 | - | - | - | 0.516 | 0.335 | 0.475 |
| Cheng 2020 [27] | Kane | 157 | - | - | - | 0.36 | - | 0.27 |
|  | Hill-RBF 2 | 157 | - | - | - | 0.37 | - | 0.26 |
|  | Holladay 1_MWK | 157 | - | - | - | 0.37 | - | 0.26 |
|  | Holladay 1_WK | 157 | - | - | - | 0.37 | - | 0.25 |
|  | SRK/T_MWK | 157 | - | - | - | 0.43 | - | 0.36 |
|  | SRK/T_WK | 157 | - | - | - | 0.42 | - | 0.33 |
|  | EVO | 157 | - | - | - | 0.39 | - | 0.29 |
|  | Barrett | 157 | - | - | - | 0.37 | - | 0.25 |
|  | Haigis | 157 | - | - | - | 0.42 | - | 0.34 |

**Table S14. Summary of studies included in the subgroup analysis (26.0 mm - 28.0 mm).**

| **Study (Author, Year)** | **Formulas** | **No. eyes** | **Percentage of IOL prediction error** | **MAE (D)** | **SD of MAE (D)** | **MedAE (D)** | **Study (Author, Year)** | **Formulas** |
| --- | --- | --- | --- | --- | --- | --- | --- | --- |
|  |  |  | **Within ± 0.25 D** | **Within ± 0.50 D** | **Within ± 1.00 D** |  |  |  |
| Wei 2020 [19] | XGBoost | 37 | - | - | - | 0.35 | 0.24 | 0.26 |
|  | Barrett | 37 | - | - | - | 0.38 | 0.27 | 0.42 |
| Lin 2021 [21] | Barrett | 49 | 42.9 | 73.5 | 100 | - | - | - |
|  | EVO | 49 | 46.9 | 71.4 | 100 | - | - | - |
|  | Haigis | 49 | 38.8 | 65 | 100 | - | - | - |
|  | Kane | 49 | 55.1 | 75.5 | 100 | - | - | - |
|  | SRK/T | 49 | 34.7 | 75 | 100 | - | - | - |
| Chen 2021 [24] | Kane | 326 | - | - | - | 0.46 | 0.42 | 0.38 |
|  | Hill-RBF 2 | 326 | - | - | - | 0.4 | 0.41 | 0.31 |
|  | Barrett | 326 | - | - | - | 0.43 | 0.29 | 0.4 |
|  | EVO | 326 | - | - | - | 0.8 | 0.97 | 0.49 |
| Ji 2019 [25] | Barrett | 20 | - | - | - | 0.32 | - | - |
|  | Hill-RBF 2 | 20 | - | - | - | 0.41 | - | - |
|  | SRK/T | 20 | - | - | - | 0.43 | - | - |
|  | Haigis | 20 | - | - | - | 0.56 | - | - |
|  | Holladay 2 | 20 | - | - | - | 0.82 | - | - |
|  | Holladay 1 | 20 | - | - | - | 1.1 | - | - |
| Wei 2022 [26] | XGBoost | 29 | - | - | - | 0.5 | 0.586 | 0.286 |
|  | Hill-RBF 3 | 29 | - | - | - | 0.448 | 0.498 | 0.225 |
|  | Kane | 29 | - | - | - | 0.533 | 0.501 | 0.315 |
|  | Barrett | 29 | - | - | - | 0.584 | 0.543 | 0.44 |
|  | EVO | 29 | - | - | - | 0.54 | 0.478 | 0.39 |
|  | K6 | 29 | - | - | - | 0.603 | 0.576 | 0.41 |
|  | Haigis WK | 29 | - | - | - | 0.575 | 0.554 | 0.41 |
|  | Haigis | 29 | - | - | - | 0.612 | 0.56 | 0.47 |
|  | SRK/T_WK | 29 | - | - | - | 0.553 | 0.528 | 0.41 |
|  | SRK/T | 29 | - | - | - | 0.485 | 0.468 | 0.41 |
| Cheng 2020 [27] | Kane | 98 | - | - | - | 0.35 | - | 0.28 |
|  | Hill-RBF 2 | 98 | - | - | - | 0.34 | - | 0.3 |
|  | Holladay 1_MWK | 98 | - | - | - | 0.34 | - | 0.3 |
|  | Holladay 1_WK | 98 | - | - | - | 0.35 | - | 0.29 |
|  | SRK/T_MWK | 98 | - | - | - | 0.39 | - | 0.3 |
|  | SRK/T_WK | 98 | - | - | - | 0.39 | - | 0.31 |
|  | EVO | 98 | - | - | - | 0.41 | - | 0.31 |
|  | Barrett | 98 | - | - | - | 0.34 | - | 0.3 |
|  | Haigis | 98 | - | - | - | 0.35 | - | 0.26 |

**Table S15. Summary of studies included in the subgroup analysis (28.0 mm - 30.0 mm).**

| **Study (Author, Year)** | **Formulas** | **No. eyes** | **Percentage of IOL prediction error** | | | **MAE (D)** | **SD of MAE (D)** | **MedAE (D)** |
| --- | --- | --- | --- | --- | --- | --- | --- | --- |
|  |  |  | **Within ± 0.25 D** | **Within ± 0.50 D** | **Within ± 1.00 D** |  |  |  |
| Wei 2020 [19] | XGBoost | 54 | - | - | - | 0.34 | 0.24 | 0.32 |
|  | Barrett | 54 | - | - | - | 0.51 | 0.3 | 0.52 |
| Lin 2021 [21] | Barrett | 45 | 44.4 | 75.6 | 97.8 | - | - | - |
|  | EVO | 45 | 46.6 | 82.2 | 100 | - | - | - |
|  | Haigis | 45 | 40 | 80 | 100 | - | - | - |
|  | Kane | 45 | 40 | 82.2 | 97.8 | - | - | - |
|  | SRK/T | 45 | 35.6 | 66.7 | 95.6 | - | - | - |
| Chen 2021 [24] | Kane | 416 | - | - | - | 0.44 | 0.37 | 0.33 |
|  | Hill-RBF 2 | 416 | - | - | - | 0.41 | 0.4 | 0.3 |
|  | Barrett | 416 | - | - | - | 0.45 | 0.31 | 0.4 |
|  | EVO | 416 | - | - | - | 0.49 | 0.52 | 0.39 |
| Ji 2019 [25] | Barrett | 17 | - | - | - | 0.3 | - | - |
|  | Hill-RBF 2 | 17 | - | - | - | 0.38 | - | - |
|  | SRK/T | 17 | - | - | - | 0.75 | - | - |
|  | Haigis | 17 | - | - | - | 0.58 | - | - |
|  | Holladay 2 | 17 | - | - | - | 1.16 | - | - |
|  | Holladay 1 | 17 | - | - | - | 1.15 | - | - |
| Wei 2022 [26] | XGBoost | 28 | - | - | - | 0.299 | 0.2 | 0.244 |
|  | Hill-RBF 3 | 28 | - | - | - | 0.316 | 0.179 | 0.34 |
|  | Kane | 28 | - | - | - | 0.599 | 0.276 | 0.603 |
|  | Barrett | 28 | - | - | - | 0.465 | 0.318 | 0.398 |
|  | EVO | 28 | - | - | - | 0.423 | 0.338 | 0.333 |
|  | K6 | 28 | - | - | - | 0.411 | 0.327 | 0.29 |
|  | Haigis WK | 28 | - | - | - | 0.443 | 0.38 | 0.393 |
|  | Haigis | 28 | - | - | - | 0.691 | 0.543 | 0.588 |
|  | SRK/T_WK | 28 | - | - | - | 0.538 | 0.409 | 0.418 |
|  | SRK/T | 28 | - | - | - | 0.675 | 0.62 | 0.528 |
| Cheng 2020 [27] | Kane | 115 | - | - | - | 0.31 | - | 0.25 |
|  | Hill-RBF 2 | 115 | - | - | - | 0.43 | - | 0.4 |
|  | Holladay 1_MWK | 115 | - | - | - | 0.31 | - | 0.29 |
|  | Holladay 1_WK | 115 | - | - | - | 0.3 | - | 0.29 |
|  | SRK/T_MWK | 115 | - | - | - | 0.34 | - | 0.31 |
|  | SRK/T_WK | 115 | - | - | - | 0.35 | - | 0.28 |
|  | EVO | 115 | - | - | - | 0.41 | - | 0.35 |
|  | Barrett | 115 | - | - | - | 0.4 | - | 0.36 |
|  | Haigis | 115 | - | - | - | 0.4 | - | 0.37 |

**Table S16. Summary of studies included in the subgroup analysis (≥ 30.0 mm).**

| **Formulas** | **26.0 mm - 28.0 mm** | | **28.0 mm - 30.0 mm** | | **≥ 30.0 mm** | |
| --- | --- | --- | --- | --- | --- | --- |
|  | **Sucra** | **PrBest** | **Sucra** | **PrBest** | **Sucra** | **PrBest** |
| Barrett | 0.58046 | 0.01785 | 0.583275 | 0.0332 | 0.488745 | 0.0044 |
| EVO | 0.35103 | 0.0046 | 0.19251 | 0.0028 | 0.44588 | 0.00925 |
| Haigis | 0.577305 | 0.10625 | 0.34957 | 0.03725 | 0.12515 | 0.00265 |
| Haigis_WK | 0.178605 | 0.00725 | 0.411315 | 0.0549 | 0.578545 | 0.058 |
| Hill_RBF_2 | 0.756005 | 0.2365 | 0.74148 | 0.3014 | 0.580605 | 0.0515 |
| Hill_RBF_3 | 0.717455 | 0.2127 | 0.644415 | 0.18995 | 0.855355 | 0.35045 |
| K6 | 0.14099 | 0.00655 | 0.36424 | 0.04275 | 0.65754 | 0.0851 |
| Kane | 0.46498 | 0.0157 | 0.583095 | 0.05085 | 0.3657 | 0.00285 |
| SRK_T | 0.51823 | 0.0656 | 0.5798 | 0.12465 | 0.15381 | 0.00465 |
| SRK_T_WK | 0.41633 | 0.03515 | 0.450665 | 0.0671 | 0.36204 | 0.0159 |
| XGBoost | 0.79861 | 0.29185 | 0.599635 | 0.09515 | 0.88663 | 0.41525 |

**Table S17. Results of network rank test in subgroup analysis.**
